# A deafness-associated mitochondrial DNA mutation caused pleiotropic effects on DNA replication and tRNA metabolism

**DOI:** 10.1093/nar/gkac720

**Published:** 2022-08-30

**Authors:** Feilong Meng, Zidong Jia, Jing Zheng, Yanchun Ji, Jing Wang, Yun Xiao, Yong Fu, Meng Wang, Feng Ling, Min-Xin Guan

**Affiliations:** Division of Medical Genetics and Genomics, The Children's Hospital, Zhejiang University School of Medicine and National Clinical Research Center for Child Health, Hangzhou, Zhejiang, China; Institute of Genetics, Zhejiang University School of Medicine, Hangzhou, Zhejiang, China; Zhejiang Provincial Key Lab of Genetic and Developmental Disorder, Hangzhou, Zhejiang, China; Division of Medical Genetics and Genomics, The Children's Hospital, Zhejiang University School of Medicine and National Clinical Research Center for Child Health, Hangzhou, Zhejiang, China; Institute of Genetics, Zhejiang University School of Medicine, Hangzhou, Zhejiang, China; Zhejiang Provincial Key Lab of Genetic and Developmental Disorder, Hangzhou, Zhejiang, China; Division of Medical Genetics and Genomics, The Children's Hospital, Zhejiang University School of Medicine and National Clinical Research Center for Child Health, Hangzhou, Zhejiang, China; Institute of Genetics, Zhejiang University School of Medicine, Hangzhou, Zhejiang, China; Zhejiang Provincial Key Lab of Genetic and Developmental Disorder, Hangzhou, Zhejiang, China; Division of Medical Genetics and Genomics, The Children's Hospital, Zhejiang University School of Medicine and National Clinical Research Center for Child Health, Hangzhou, Zhejiang, China; Institute of Genetics, Zhejiang University School of Medicine, Hangzhou, Zhejiang, China; Zhejiang Provincial Key Lab of Genetic and Developmental Disorder, Hangzhou, Zhejiang, China; Institute of Genetics, Zhejiang University School of Medicine, Hangzhou, Zhejiang, China; Institute of Genetics, Zhejiang University School of Medicine, Hangzhou, Zhejiang, China; Division of Otolaryngology-Head and Neck Surgery, The Children's Hospital, Zhejiang University School of Medicine, Hangzhou, Zhejiang, China; Division of Medical Genetics and Genomics, The Children's Hospital, Zhejiang University School of Medicine and National Clinical Research Center for Child Health, Hangzhou, Zhejiang, China; Institute of Genetics, Zhejiang University School of Medicine, Hangzhou, Zhejiang, China; Zhejiang Provincial Key Lab of Genetic and Developmental Disorder, Hangzhou, Zhejiang, China; Chemical Genomics Research Group, RIKEN Center for Sustainable Resource Science, Hirosawa 2-1, Wako, Saitama, Japan; Division of Medical Genetics and Genomics, The Children's Hospital, Zhejiang University School of Medicine and National Clinical Research Center for Child Health, Hangzhou, Zhejiang, China; Institute of Genetics, Zhejiang University School of Medicine, Hangzhou, Zhejiang, China; Zhejiang Provincial Key Lab of Genetic and Developmental Disorder, Hangzhou, Zhejiang, China; Joint Institute of Genetics and Genome Medicine between Zhejiang University and University of Toronto, Hangzhou, Zhejiang, China

## Abstract

In this report, we investigated the molecular mechanism underlying a deafness-associated m.5783C > T mutation that affects the canonical C50-G63 base-pairing of TΨC stem of tRNA^Cys^ and immediately adjacent to 5′ end of light-strand origin of mitochondrial DNA (mtDNA) replication (OriL). Two dimensional agarose gel electrophoresis revealed marked decreases in the replication intermediates including ascending arm of Y-fork arcs spanning OriL in the mutant cybrids bearing m.5783C > T mutation. mtDNA replication alterations were further evidenced by decreased levels of PolγA, Twinkle and SSBP1, newly synthesized mtDNA and mtDNA contents in the mutant cybrids. The m.5783C > T mutation altered tRNA^Cys^ structure and function, including decreased melting temperature, conformational changes, instability and deficient aminoacylation of mutated tRNA^Cys^. The m.5783C > T mutation impaired the 5′ end processing efficiency of tRNA^Cys^ precursors and reduced the levels of tRNA^Cys^ and downstream tRNA^Tyr^. The aberrant tRNA metabolism impaired mitochondrial translation, which was especially pronounced effects in the polypeptides harboring higher numbers of cysteine and tyrosine codons. These alterations led to deficient oxidative phosphorylation including instability and reduced activities of the respiratory chain enzyme complexes I, III, IV and intact supercomplexes overall. Our findings highlight the impact of mitochondrial dysfunction on deafness arising from defects in mitochondrial DNA replication and tRNA metabolism.

## INTRODUCTION

Mutations in mitochondrial DNA (mtDNA) have been associated with a wide spectrum of human diseases including neuromuscular disorders, diabetes, vision failure as well as hearing loss (1–5). Human mitochondrial DNA (mtDNA) is circular, double-strand molecule with 16.6 kb and composed of heavy (H)- strand encoding 2 rRNAs and 14 tRNAs and 12 polypeptides for essential subunits of oxidative phosphorylation system (OXPHOS), and light (L)- strand coding for eight tRNAs and one polypeptide (ND6) (6,7). Human mitochondrial genome contains two conserved origins of DNA replication (heavy-strand origin, OriH, at positions 110–441 at D-loop region, and light-strand origin, OriL at positions 5721–5798 containing the 37 bp at 3′ end anti-sense strand of tRNA^Cys^ gene, 32 bp noncoding sequence and 9 bp of 5′ end anti-sense strand of tRNA^Asn^ gene) (8–10). The strand-displacement model (SDM) and alternative and partially overlapping models have been suggested for mtDNA replication, catalyzed by own replication machinery including DNA polymerase γ (Polγ), Twinkle helicase and single stranded DNA binding protein (mtSSB) (8–11). mtDNA bi-directionally produces the polycistronic H- and L- strand transcripts, catalyzed by the mitochondrial transcription machinery and resultant polycistronic transcripts are processed to release 13 mRNAs, 22 tRNAs and 2 rRNAs essential for mitochondrial translation (12–15). In particular, the processing of mitochondrial tRNAs from the primary transcripts required the precise cleavage of tRNAs at their 5′ ends, catalyzed by RNase P, and 3′ terminal mediated by RNase Z, respectively (16–18). The defects in mitochondrial RNA metabolism have been linked to sensorineural hearing loss that often occurs as a consequence of damaged or deficient inner ear hair cells (5,19). The deafness-associated 12S rRNA 1555A > G and 1494C > T mutations at the A site of ribosomes make the human mitochondrial ribosomes more bacteria-like and alter binding sites for aminoglycosides and mitochondrial translation (20–22). These deafness-linked tRNA mutations such as m.7516DelA and m.4295A > G impacted their structure and functions including the processing of the tRNAs from the primary transcripts, stability of folded secondary structure, charging of tRNA, or codon-anticodon interaction in the process of mitochondrial translation (23–32). However, the pathogenic mechanisms underlying defects in mitochondrial RNA metabolism still remain elusive.

**Figure 1.**
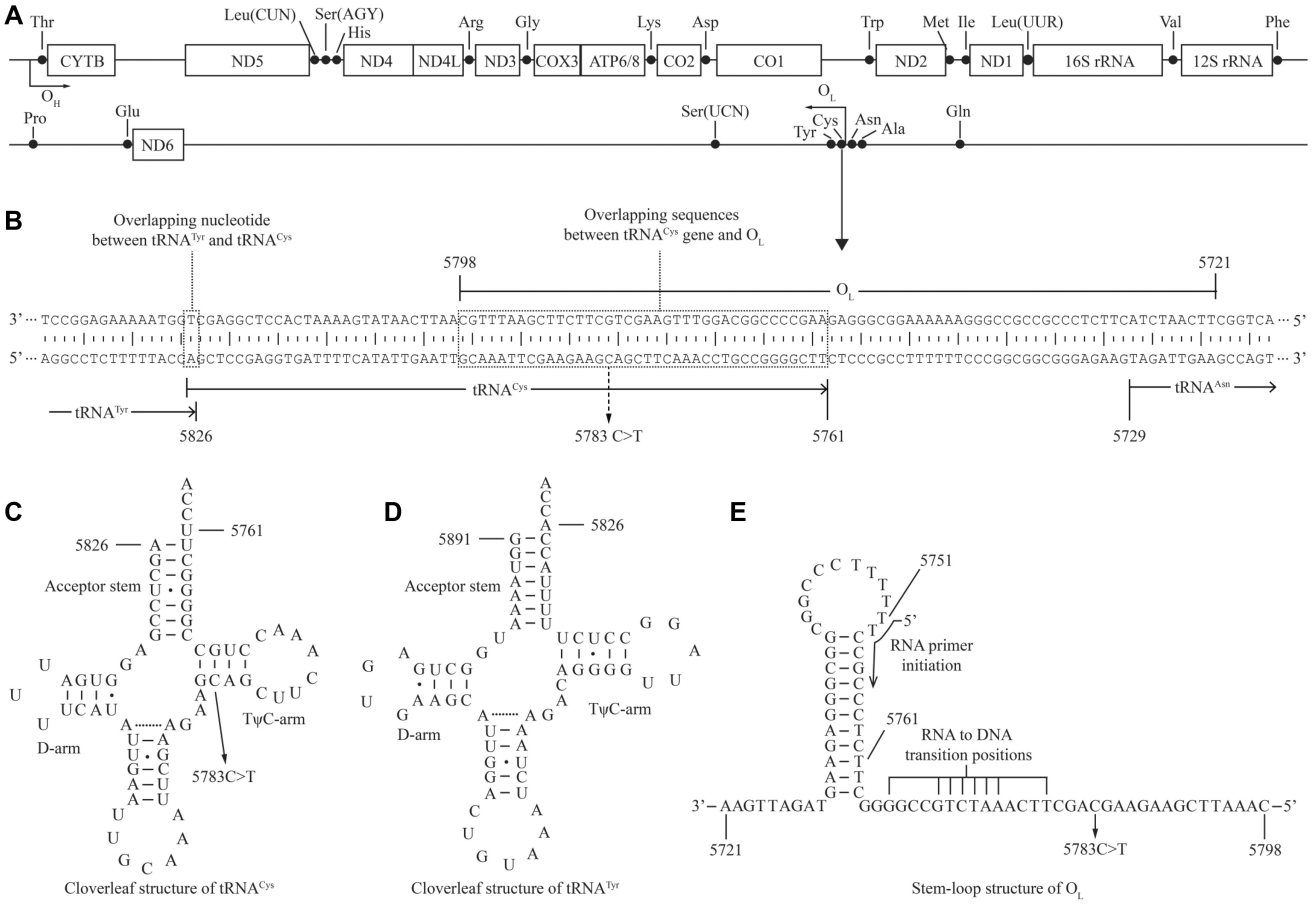
A schema of genome map of human mitochondria and location of m.5783C > T mutation. (**A**) H-strand encodes 12S rRNA, 16S rRNA, 12 mRNAs and 14 tRNAs and L-strand codes for 8 tRNAs and ND6 mRNA. The heavy strand origin (O_H_) is located in the strand-displacement loop (D-loop) and the origin for the light strand (O_L_) is located in a cluster of tRNAs, two-thirds of the genome away. (**B**) The O_L_ at position 5721–5798 (37 bp at 3′ end anti-sense strand of tRNA^Cys^ gene, 32 bp noncoding sequence and 9 bp of anti-sense strand of tRNA^Asn^ gene) and adjacent sequences. The adenine at position 5826 is a sharing nucleotide that acts as the 5′end of tRNA^Cys^ and 3′end of tRNA^Tyr^ genes, respectively. (**C**, **D**) Cloverleaf structures of tRNA^Cys^ and tRNA^Tyr^ were derived from Florentz *et al.* (2003) (33). **(E)** Stem-loop structure of O_L_ and adjacent sequences required for L-strand replication (34,35). The m.5783C > T mutation, RNA primer initiation and RNA to DNA transition sites are indicated.

Most recently, a homoplasmic 5783C > T mutation in mitochondrial tRNA^Cys^ gene was identified in one Han Chinese family with maternal inheritance of deafness from a large cohort of Chinese hearing-impaired probands (32). As shown in Figure 1, the m.5783C > T mutation locates at a highly conserved cytosine at position 50 (C50) of TΨC stem of tRNA^Cys^ and immediately adjacent to 5′ end of OriL at positions 5721–5798 containing the 37 bp of 3′ end anti-sense strand of tRNA^Cys^ gene (33–36). Unlike only impacts of other deafness-linked mtDNA mutations on tRNA structure and function (23–31), the m.5783C > T mutation may cause pleiotropic effects on mitochondrial DNA replication and tRNA metabolism. The effects of the m.5783C > T mutation on mtDNA replication were evaluated by neutral two dimensional agarose gel electrophoresis technique in the cybrids, generated by transferring mitochondria from lymphoblastoid cell lines from one Chinese family into mtDNA-less *ρ*^o^206 cells (37–39). The method involves separating restriction enzymes digested replication intermediates of different mtDNA mass and hybridizing with probes to allow an examination of replication intermediates in specific mtDNA regions (40). The change of C50-G63 base-pairing with U50-G63 base-pairing at TΨC stem of tRNA^Cys^ by m.5783C > T mutation may perturb the structure and function of tRNA^Cys^ and subsequently impair mitochondrial translation and oxidative phosphorylation. The impact of m.5783C > T mutation on tRNA^Cys^ structure and function was assessed for the processing of tRNA precursors, stability and aminoacylation of mitochondrial tRNAs. These cell lines were further examined for the effect of the m.5783C > T mutation on the mitochondrial protein synthesis, stability and activities of oxidative phosphorylation system.

## MATERIALS AND METHODS

### Subjects and audiological examinations

One hearing-impaired Chinese Han pedigree for this study was ascertained at the Otology Clinic of the Children's Hospital, Zhejiang University School of Medicine, as described previously (32). This study was in compliance with the Declaration of Helsinki. Informed consent, blood samples and clinical evaluations were obtained from all participants and families, under protocols approved by Ethic Committees of Zhejiang University School of Medicine. Audiological and neurological examinations of hearing impairment were performed as detailed previously (41). All available members of the pedigree and control subjects were evaluated at length to identify both personal and family medical history of hearing loss, the history of the use of aminoglycosides and other clinical abnormalities.

### Analysis of mitochondrial DNA

Genomic DNA was isolated from whole blood of participants using QIAamp DNA Blood Mini Kit (Qiagen, No. 51104). The subject's DNA fragments spanning the tRNA^Cys^ gene were PCR amplified by use of oligodeoxynucleotides corresponding to mtDNA at positions 5751–5843 and then analyzed by direct sequencing (42). Sanger sequence analyses of entire mtDNAs of proband WZD117 III-7 and one Chinese control subject (C62) were undertaken as described previously (43). The presence and amount of m.5783C > T mutation from members of the Chinese pedigree and derived cell lines were examined as detailed elsewhere (42). These sequence results were compared with the updated consensus Cambridge sequence (GenBank accession number: NC 012920) (6).

mtDNA contents in various cell lines were measured with Southern blot and quantitative real-time PCR (qPCR) analyses, as detailed elsewhere (44). For Southern blotting analysis, 1 μg of genomic DNA was digested with *BamH*I, run on a 1% agarose gel and then electroblotted onto a positively charged nylon membrane (Roche) for the hybridization analysis with digoxigenin (DIG)-labeled oligodeoxynucleotide probes. Probes were synthesized by PCR DIG Probe Synthesis Kit (Roche) with following primers: D-loop 5′-TACGTTCAATATTACAGGCGAAC-3′ (sense) and 5′-TTGCTTTGAGGAGGTAAGCTAC-3′ (anti-sense); 18S rRNA 5′-TACCTGGTTGATCCTGCCAG-3′ (sense) and 5′-TCGGGAGTGGGTAATTTGC-3′ (anti-sense), respectively.

For qPCR assays, 50 ng of genomic DNA from various cell lines were used in each reaction using Universal SYBR Green Master (Roche Applied Science, Mannheim, Germany) with primers for mtDNA (300 nM each) in a 7900 HT Fast Real-time PCR System (Applied Biosystems) and for *β*-actin for normalization. The following primers were used: mtDNA 5′-TCACCCTATTAACCACTCA-3′ (sense) and 5′-AGACAGATACTGCGACATA-3′ (anti-sense); *β*-actin 5′-TCACCCACACTGTGCCCATCTACGA-3′ (sense) and 5′-CAGCGGAACCGCTCATTGCCAATGG-3′ (antisense). mtDNA contents were calculated using the ΔΔCt method whereby all D-loop (mtDNA target) Ct values were first normalized to *β*-actin. Data from multiple experiments were analyzed using the procedure as described elsewhere (44).

### Cell lines and culture conditions

Immortalized lymphoblastoid cell lines were generated from one hearing-impaired proband (III-7) carrying the m.5783C > T mutation and one genetically unrelated Chinese control individual (C62) belonging to the same mtDNA haplogroup H2 but lacking the mutation. These cell lines were grown in RPMI 1640 medium with 10% fetal bovine serum (FBS). The bromodeoxyuridine (BrdU) resistant 143B.TK^−^ cell line was grown in Dulbecco's modified Eagle's medium (DMEM) (Life Technologies) (containing 4.5 mg of glucose and 0.11 mg pyruvate/ml), supplemented with 100 μg of BrdU/ml and 5% FBS. The mtDNA-less *ρ*^o^206 cell line, derived from 143B.TK^−^ was grown under the same conditions as the parental line, except for the addition of 50 μg of uridine/ml. Transformation by cytoplasts of mtDNA-less *ρ*^o^206 cells using one affected subject (III-7) carrying the m.5783C > T mutation and one control individual (C62) was performed as described elsewhere (37–39). All cybrid cell lines constructed with enucleated lymphoblastoid cell lines were maintained in the same medium as the 143B.TK^−^ cell line. Three mutant cybrids (III-7.5, III-7.6 and III- 7.9) carrying the m.5783C > T mutation and three control cybrids (C62.4, C62.6 and C62.7) lacking the mutation with similar mtDNA copy numbers and the same karyotype were used for the biochemical characterization described below.

### Analysis of mtDNA replication

Analysis of mtDNA replication by neutral 2D agarose gel electrophoresis was carried out as detailed previously (9,40). Briefly, mtDNAs were obtained from mitochondria isolated from cybrids by phenol/chloroform extraction. Eight micrograms of mtDNAs from various cell lines were digested with *Acc*I, *Dra*I, *Bcl*I and *Apa*I, respectively, and loaded on 0.4% agarose gel (1D gel), and run at 8 V for 24 h at room temperature. The DNA-containing lanes were cut, rotated 90° and casted around the gel slices with 1% agarose containing 0.5 mg/ml ethidium bromide (EtBr) (2D gel). 2D gels were run at 120 V for 4 h at 4°C and then electroblotted onto a positively charged nylon membrane (Roche) for the hybridization analysis with DIG-labeled oligodeoxynucleotide probes a, b, c and d (fragments corresponding to mtDNA positions 16 343–150, 3120–3560, 5450–5932 and 10 436–11 601), respectively (9).

Southwestern analysis for measurement of the nascent mtDNA in cell lines were performed as described previously (45). Nascent mtDNA was labeled by incubating cybrids cells with 10 μM of BrdU in fresh medium for 24 h. Total genomic DNAs were extracted and processed as for Southern blots, except post-transfer, the membranes were blocked with 10% BSA and probed with anti-BrdU antibody (Proteintech, 66241-1-lg) to detect newly synthesized mtDNA. After probing for incorporated BrdU, the membrane was further probed with a DIG-labeled 18S rRNA probe as described above.

### UV melting assays

UV melting assays were carried out as described elsewhere (29,46). The wild type and mutant tRNA^Cys^ transcripts were generated by *in vitro* transcription by T7 RNA polymerase (Promega) using synthetic DNA oligonucleotides as templates. The sequences of oligonucleotides were 5′- TAATACGACTCACTATAAGCTCCGAGGTGATTTTCATATTGAATTGCAAATTCGAAGAAGCAGCTTCAAACCTGCCGGGGCTT-3′ (wild type);

5′TAATACGACTCACTATAAGCTCCGAGGTGATTTTCATATTGAATTGCAAATTCGAAGAAGTAGCTTCAAACCTGCCGGGGCTT-3′ (mutant). The tRNA^Cys^ transcripts were diluted in the buffer including 50 mM sodium phosphate (pH 7.0), 50 mM NaCl, 5 mM MgCl_2_ and 0.1 mM EDTA. Absorbance against temperature melting curves were measured at 260 nm with a heating rate of 1°C /min from 25 to 95°C through Agilent Cary 100 UV Spectrophotometer.

### Mitochondrial RNase P and RNase Z cleavage assays

The wild type and mutant precursors of tRNA^Cys^ corresponding to mtDNA at positions 5862 (5′) to 5761 (3′) for 5' end processing assay or 5826 (5′) to 5725 (3′) for 3' end processing assay were cloned into the pCRII-TOPO vector carrying SP6 and T7 promoters (Clonetech). After *Hind*III digestion, the RNA substrates (105 nt) were transcribed with T7 RNA polymerase, in the presence of 10 μM ATP, CTP, GTP and UTP, pH 7.5 and 10 units RNase inhibitor at 20°C. Transcripts were purified by denaturing polyacrylamide gel electrophoresis (PAGE) (8 M urea, 8% polyacrylamide/bisacrylamide [19:1]) and were dissolved in 1 mM EDTA. Mitochondrial RNase P was reconstituted from purified recombinant proteins MRPP1, MRPP2 and MRPP3 as described previously (17). The reaction mixtures were incubated in 40 μl of reaction buffer containing 20 mM HEPES (pH 7.6), 20 mM KCl, 2 mM MgCl_2_, 2mM DTT, 0.1 mg/ml bovine serum albumin, 80 μM S-adenosyl methionine, 1 U RiboLock RNase Inhibitor (Thermo Fisher Scientific), 300 ng pre-tRNAs, 800 nM MRPP1/2. The reaction mixes were pre-incubated at 30°C for 15 min, and the reaction was initiated by addition of MRPP3 and pre-tRNA substrates with subsequent incubation at 30°C. After 5, 10, 15, 20 and 25 min, 5 μl of aliquots were withdrawn and stopped by addition of 5 μl loading buffer (85% formamide, 10 mM EDTA). Mitochondrial RNase Z was reconstituted from purified recombinant proteins ELAC2 as detailed elsewhere (16,47). The reaction procedures were the same as RNase P, except incubated in assay buffer with 800 nM ELAC2 instead of MRPP3. Reaction products were resolved via denaturing PAGE, then electroblotted onto a nylon membrane (Roche) and hybridized with DIG-labeled oligodeoxynucleotide probes for tRNA^Cys^ precursors. DIG-labeled probes were generated by using DIG-oligonucleotide Tailing kit (Roche). The hybridization and quantification of density in each band were performed as detailed previously (25).

### Mitochondrial tRNA analysis

Total mitochondrial RNAs were obtained from mitochondria isolated from various cybrid cell lines (∼2.0 × 10^7^ cells), as described previously (48). The tRNA Northern blot analysis was performed as detailed elsewhere (46). DIG-labeled oligodeoxynucleotide probes specific for 22 mitochondrial tRNAs and 5S rRNA were as detailed elsewhere (15). The hybridization and quantification of density in each band were carried out as detailed previously (46).

For the aminoacylation assays, total mitochondrial RNAs were isolated under acid conditions, and 4 μg of total mitochondrial RNAs were electrophoresed at 4°C through an acid (pH 5.0) 10% polyacrylamide-8 M urea gel to separate the charged and uncharged tRNA as detailed elsewhere (46,49). To further distinguish nonaminoacylated tRNA from aminoacylated tRNA, samples of tRNAs were deacylated by being heated for 10 min at 60°C at pH 8.3 and then run in parallel (46,49). The gels were then electroblotted onto a positively charged nylon membrane (Roche) for the hybridization analysis with oligodeoxynucleotide probes as described above. Quantification of density in each band was performed as detailed previously (46).

For the tRNA mobility shift assay, 2 μg of total mitochondrial RNAs were electrophoresed through a 10% polyacrylamide native gel at 4°C with 50 mM Tris–glycine buffer. After electrophoresis, the gels were treated according to the Northern blot analysis as described above (29,46).

tRNA half-life measurements were performed as detailed elsewhere (50). Briefly, various cell lines were incubated in the DMEM medium, supplemented with 5% FBS and 250 ng/ml EtBr for 4, 6, 8, 24 h, respectively. Ten micrograms of total cellular RNAs, extracted as above, were subjected to Northern blot tRNA analysis as detailed above.

### Western blot analysis

Western blot analysis was performed as detailed previously (26). Twenty micrograms of total cell proteins obtained from various cell lines were denatured and loaded on sodium dodecyl sulfate (SDS) polyacrylamide gels. The gels were electroblotted onto a polyvinylidene difluoride (PVDF) membrane for hybridization. The antibodies used for this investigation were from Abcam [TOM20 (ab56783), Total OXPHOS Human WB Antibody Cocktail (ab110411), Proteintech [*β*-actin (20536-1-AP), Twinkle (13435-1-AP), SSBP1 (12212-1-AP), ABclonal [PolγA (A1323), ELAC2 (A7128), TRNT1 (A17699), MRPP2 (A0959)], Invitrogen [POLRMT (PA5-28196)] and BOSTER [TFAM (BA2827)]. Peroxidase AffiniPure goat anti-mouse IgG and goat anti-rabbit IgG (Jackson) were used as secondary antibodies and protein signals were detected using the ECL system (CW- BIO). Quantification of density in each band was performed as detailed elsewhere (26).

### Mitochondrial translation assays

Pulse-labeling of the mutant and control cell lines for 30 min with [^35^S] methionine-[^35^S] cysteine in methionine-free DMEM in the presence of emetine, electrophoretic analysis of the translation products, and quantification of radioactivity in the whole-electrophoretic patterns or in individual well-resolved bands were carried out as detailed previously (39,51).

### Blue native gel electrophoresis and assays of activities of respiratory chain complexes

Blue native gel electrophoresis (BN-PAGE) and in-gel activity assay were performed by using mitochondrial proteins isolated from mutant and control cybrid cell lines, as detailed elsewhere (52–54). The enzymatic activities of respiratory chain complexes I, II, III and IV from various cell lines were assayed as described previously (54,55).

### Statistical analysis

Statistical analysis was carried out using the unpaired, two-tailed Student's *t*-test contained in the Microsoft-Excel program or Macintosh (version 2019). Differences were considered significant at a *P* < 0.05.

## RESULTS

### Clinical presentations and derived cell lines of a hearing-impaired Chinese pedigree

One Han Chinese hearing-impaired proband (WZD117-III-7) carrying the m.5783C > T mutation was identified among 2651 Chinese hearing-impaired probands but absent in 574 Chinese normal hearing controls (32). He suffered from severe hearing loss (78 dB and 79 dB at right and left ears, respectively; a slop-shaped pattern) at the age of 23 year old. Clinical evaluations of members of the Chinese pedigree exhibited the maternal inheritance of hearing loss (Supplemental Figure S1). Five of 13 matrilineal relatives exhibited a variable degree of hearing impairment (two with moderate hearing loss and three with severe hearing loss), whereas none of the other members in this family had hearing loss (Supplemental Figure S2 and Supplemental Table S1). The age-at-onset of hearing loss ranged from 20 to 46 years, with an average of 32 year old. There was no evidence that any of the other members of this family had any other causes to account for hearing loss. These matrilineal relatives showed no other clinical presentations. Further analysis showed that the m.5783C > T mutation was present in homoplasmy in all matrilineal relatives but not in other members of this family (Supplemental Figure S1B, C).

Immortalized lymphoblastoid cell lines were derived from one affected matrilineal relative carrying the m.5783C > T mutation (III-7; male, 26 years) and from one genetically unrelated control subject (C62, female, 25 years) lacking the m.5783C > T mutation belonging to the same mtDNA haplogroup H2 (Supplemental Table S2). Cybrids were generated by transferring mitochondria from lymphoblastoid cell lines into mtDNA-less *ρ*^o^206 cells (37–39). These cybrid clones were subsequently analyzed for the presence and levels of the m.5783C > T mutation (42). After culturing for two weeks, three mutant cybrids and three control cybrids with similar mtDNA copy numbers and the same karyotype (Supplemental Figure S3) were used for the biochemical characterization described below.

### Defects in mtDNA replication

**Figure 2.**
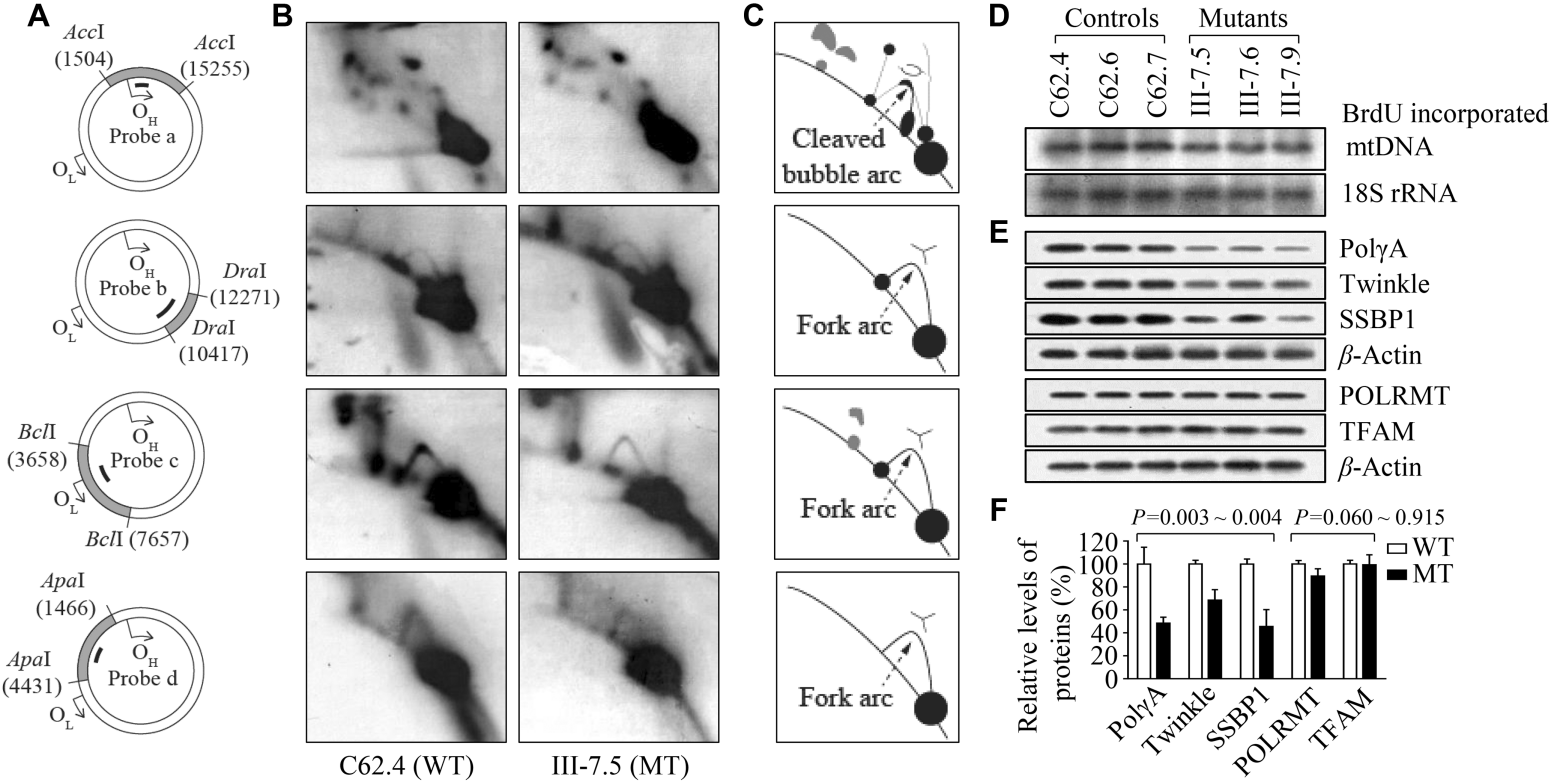
mtDNA replication analysis. (**A**) Schematic representation of fragments digested by *Acc*I, *Dra*I, *Bcl*I and *Apa*I, and hybridized with probes *a*, *b*, c and d, respectively. (**B**) Replication intermediates in specific DNA regions. Eight micrograms of mtDNAs from various cell lines were digested with *Acc*I, *Dra*I, *Bcl*I and *Apa*I, and the resultant fragments were separated in a 0.4% agarose gel. A gel slice was then cut, embedded into a 1% agarose gel and subjected to a second-dimension gel electrophoresis. The 2D gel is then blotted and hybridized with DIG labeled probes *a*, *b*, c and d, respectively. (**C**) Schematic representation of the cleaved replication bubble and Y arc formed by mtDNA replication intermediates. (**D**) South-western analysis of mtDNA replication. BrdU incorporation into mtDNA was analyzed 24 h post-injection and bands were quantified from the same blot. The nuclear 18S rRNA gene was probed to control for loading. Representative bands are shown for clarity of comparison. (**E**) Western blot analysis of proteins related to mtDNA replication: PolγA, Twinkle, SSBP1, POLRMT and TFAM with *β*-actin as a loading control in the mutant cell lines and control cell lines. (**F**) Quantification of proteins. Average relative values of PolγA, Twinkle, SSBP1, POLRMT and TFAM were normalized to the average values of *β*-actin in various cell lines. The values for the latter are expressed as percentages of the average values for the control cell lines. The average of three independent determinations for each cell line is shown. The error bars indicate two standard deviations. *P* indicates the significance, according to the *t*-test, of the differences between mutant and control cell lines.

In the SDM model, replication initiates at OriH and continues around approximately two-thirds of the genome (∼11 kb) until reaching OriL, whereby L-strand synthesis commences in the opposite direction resulting in unidirectional fork progression from each distinct origin (8–11). We used neutral 2D agarose gel electrophoresis technique to investigate the effect of m.5783C > T mutation on mtDNA replication by examining replication intermediates in specific mtDNA regions. As shown in Figure 2, equal amounts (8 μg) of mtDNAs isolated from mutant and control cybrids after culturing for two weeks were digested with *Acc*I, *Dra*I, *Bcl*I and *Apa*I, subjected to 2D agarose gel electrophoresis and hybridized them with DIG-labeled oligodeoxynucleotide probes *a*, *b*, c and d, respectively. As shown in Figure 2B and C, 2.8 kb *Acc*I fragment (nt 15 255−1504) spanning OriH and 1.85 kb *Dra*I fragment (nt 10 417–12 271) revealed similar patterns of mtDNA replication intermediates that formed archetypal bubble arcs in the *Acc*I fragment and fork arcs in the *Dra*I fragment between mutant and control cybrids. The mutant cybrids exhibited significant reductions in the replication intermediates including ∼66% (*P* < 0.001) decreases in the ascending arm of Y-fork arcs in 4 kb *Bcl*I fragment (nt 3658–7657) spanning OriL and 22% (*P* = 0.009) reductions in the fork arcs in the 3 kb *Apa*I fragment (nt 1446–4431), as compared with those in the control cybrids.

We assessed the amount of newly synthesized mtDNA in mutant and control cybrids using Southwestern blot analysis in the presence BrdU. In fact, BrdU, an analog of the nucleoside thymidine, is incorporated into replicating DNA and can be detected using anti-BrdU antibodies (45). In this assay, BrdU is only incorporated into the mitochondrial but not nuclear DNA in these cybrids, which was thymidine kinase 1 deficient. The labeled mtDNA was probed with anti-BrdU antibody and then hybridized with nucleus-encoding 18S rRNA as the loading control. As shown in Figure 2D, the nascent BrdU-labeled mtDNA levels in the mutant cybrids were decreased as compared to those in control cybrids. For comparison, the average levels of BrdU-labeled mtDNA in the various cybrids were normalized to the average levels of 18S rRNA in the same cell lines for reference. Three mutant cybrid cell lines showed average 15% less BrdU incorporation than did three control cybrids, indicating slower mtDNA replication in mutant cybrids.

We then examined the levels of components of mitochondrial replisome complex [PolγA, Twinkle, SSBP1], POLRMT (RNA polymerase) and TFAM (mitochondrial transcription factor A) using Western blot analysis (10,14). As shown in Figure 2E and F, the mutant cell lines revealed marked decreases in the levels of PolγA (61.5%), Twinkle (31.4%) and SSBP1 (54.4%), as compared with those in the control cell lines. However, the levels of POLRMT and TFAM in mutant cell lines were comparable with those in control cell lines. These data further support the impact of m.5783C > T mutation on the mtDNA replication.

### Decreases in the mtDNA contents

**Figure 3.**
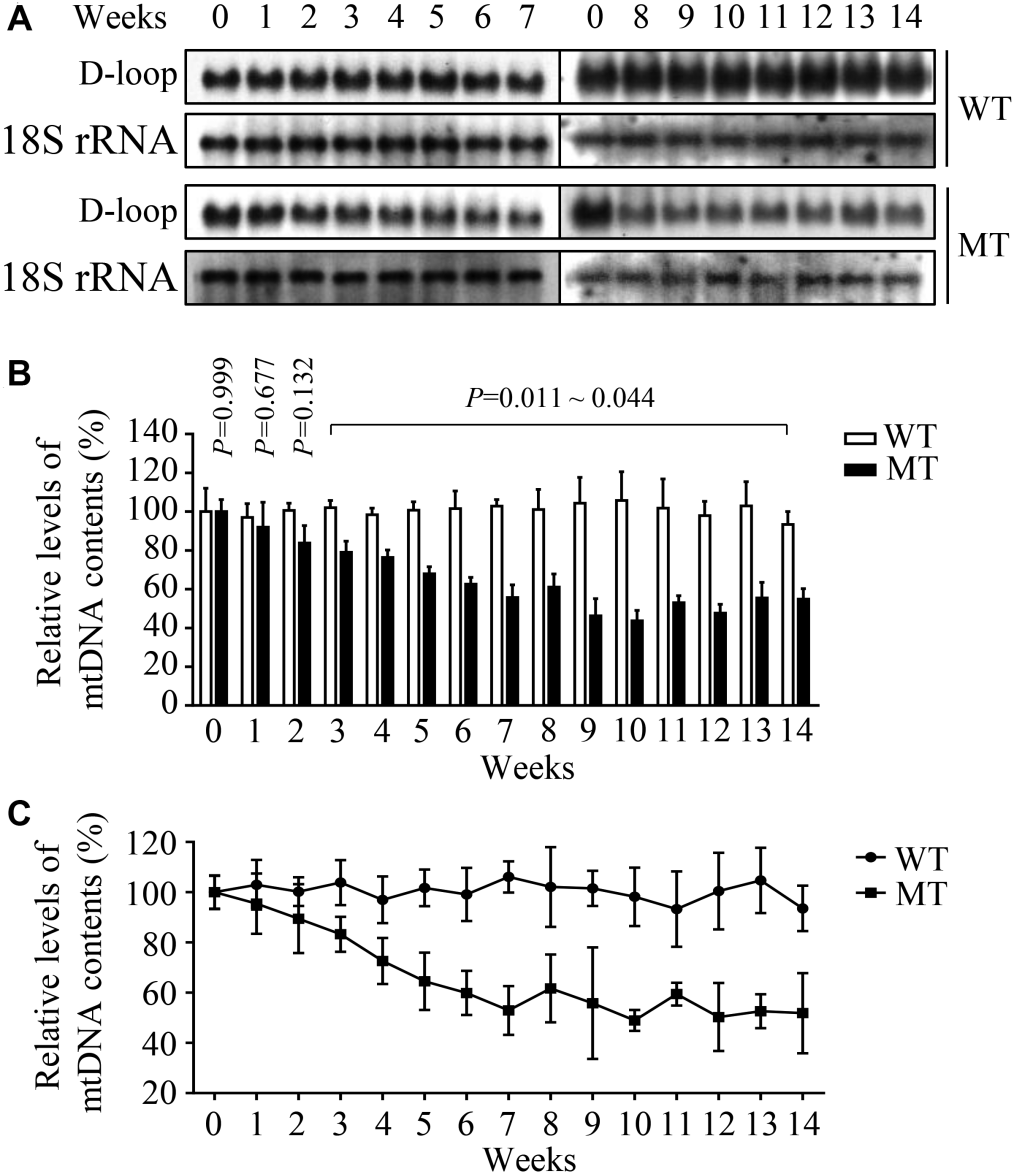
Quantification of mtDNA contents. (**A**) Southern blot analysis of mtDNA. Equal amounts (1 μg) of genomic DNA from mutant and control cybrids after culturing from time 0 to 14 weeks were digested with *BamH* I, fractionated by electrophoresis through a 1.8% agarose gel, transferred onto a positively charged membrane, and hybridized with DIG-labeled probes specific for mtDNA D-Loop region and 18S rRNA as an internal control. (**B**) Quantification of mtDNA contents. Average relative mtDNA contents per cell were normalized to the average contents per cell of 18S rRNA in mutant and control cybrids. Relative mtDNA contents were expressed as percentages of the value for various weeks of those taken at zero time in each gel. The calculations were based on three independent experiments. (**C**) Measurement of mtDNA contents by qPCR. Mitochondrial DNAs from mutant and control cybrids after culturing from time 0 to 14 weeks were normalized to *β*-actin encoded by nuclear gene. The calculations were based on three independent experiments. Graph details and symbols are explained in the legend to Figure 2.

To further investigate the effect of m.5783C > T mutation on mtDNA replication, we measured mtDNA contents in the various cell lines using Southern blot and qPCR assays. We performed Southern blot analysis by isolating total DNA from mutant and control cybrids after culturing from 1 to 14 weeks, digesting with restriction enzyme *BamH*I, separating them by electrophoresis, blotting, and hybridizing with DIG-labeled probes specific for D-loop region and 18S rRNA as the normalization (56). As shown in Figure 3A, the levels of mtDNA contents in mutant cybrids exhibited progressive decreases during culturing first 7 weeks, but not further reductions until 14 weeks, as compared with control cybrids. For comparison, the average amount of mtDNA in the various cell lines were normalized to the average levels of 18S rRNA in the same cell lines for reference. Relative mtDNA contents were expressed as percentages of the value for various weeks of day 0 in each gel. As shown in Figure 3B, the average levels of mtDNA in three mutant cell lines cultured from 1 to 14 weeks were 91.9%, 83.8%, 79.0%, 76.3%, 67.9%, 62.6%, 55.6%, 61.1%, 46.3%, 43.7%, 53.1%, 47.6%, 55.5% and 54.9% related to the mean values of those in three control cybrids, respectively (Figure 3B).

We then examined the mtDNA contents by quantitative PCR using total genomic DNA as template. In particular, mtDNA contents were measured using DNA segments spanning the D-loop region as mtDNA targets and *β*-actin as the normalization. As shown in Figure 3C, the average mtDNA contents in three mutant cybrids cultured from 1 to 14 weeks were 95.4%, 89.4%, 83.2%, 72.6%, 64.5%, 59.9%, 52.9%, 61.6%, 55.8%, 48.9%, 59.4%, 50.3%, 52.6% and 51.8% related to average values of three control cybrids, respectively. These results indicated that m.5783C > T mutation affected mtDNA contents.

### Alterations in the stability and conformation of tRNA^Cys^

**Figure 4.**
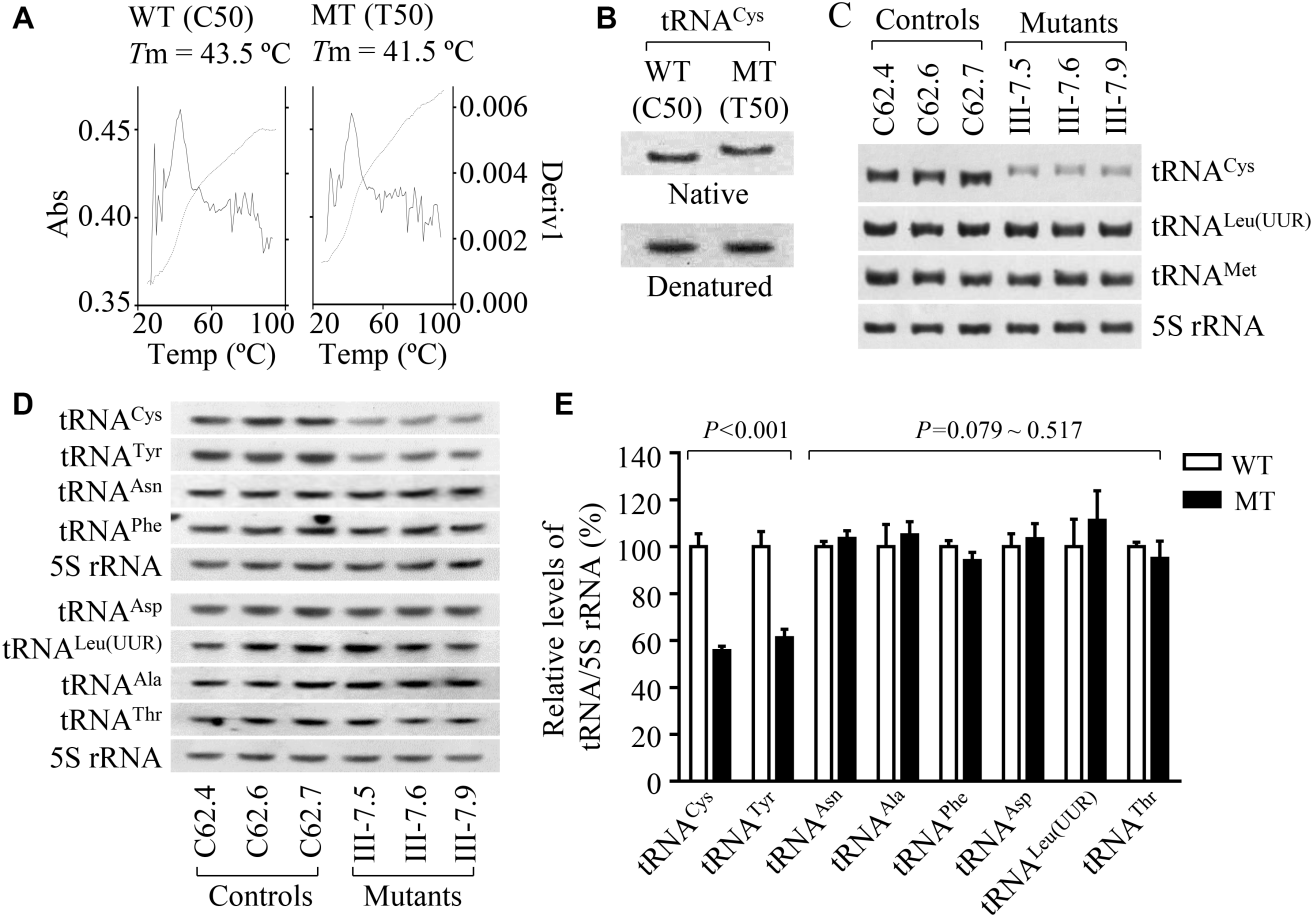
Analysis of conformation and stability of tRNA^Cys^. (**A**) Thermal stability of wild type (C50) and mutant (T50) tRNA^Cys^ transcripts. Melting profiles of WT and MT tRNA^Cys^ transcripts measured at 260 nm with a heating rate of 1°C/min from 25 to 95°C (dotted lines). First derivative (dA/dT) against temperature curves were shown to highlight the *T*m value transitions (solid lines). (**B**) Assessment of conformation changes by PAGE analysis of tRNA^Cys^ transcripts under denaturing and native conditions. The transcripts of WT and MT tRNA^Cys^ were electrophoresed through native or denaturing polyacrylamide gel, electroblotted, and hybridized with DIG-labeled oligonucleotide tRNA^Cys^ probe. (**C**, **D**) Northern blot analysis of tRNAs under native or denaturing conditions. Two micrograms of total mitochondrial RNA from various cell lines were electrophoresed through polyacrylamide gel, electroblotted, and hybridized with DIG-labeled oligonucleotide tRNA probes for: (C) tRNA^Cys^, tRNA^leu(UUR)^ and tRNA^Met^ under native condition. (**D**) tRNA^Cys^, tRNA^Tyr^, tRNA^Asn^, tRNA^Phe^, tRNA^Asp^, tRNA^Leu(UUR)^, tRNA^Ala^, tRNA^Thr^ and 5S rRNA under denaturing conditions. (**E**) Quantification of tRNA levels under denaturing condition. Average relative tRNAs content per cell were normalized to the average content per cell of 5S rRNA in three mutant cybrids and three control cybrids. The values for the latter are expressed as percentages of the average values for the control cybrids. Graph details and symbols are explained in the legend to Figure 2.

It was hypothesized that the m.5783C > T mutation alters the structure and function of tRNA^Cys^. The stability of transcripts of wild-type and mutant tRNA^Cys^ was evaluated by the measurement of the melting temperatures (*T*m) by calculating the derivatives of absorbance against a temperature curve. As shown in Figure 4A, the *T*m values for wild-type (C50) and mutant (T50) transcripts were 43.5 ± 0.2°C and 41.5 ± 0.5°C, respectively. This suggested that the m.5783C > T mutation affected the stability of tRNA^Cys^. These transcripts were then assessed for conformation change by PAGE analysis under denaturing and native conditions. As shown in Figure 4B, the wild-type (C50) tRNA^Cys^ transcript migrated slightly faster than the mutant (U50) tRNA^Cys^ transcript under the native condition, while there was no migration difference between wild-type (C50) and mutant (U50) tRNA^Cys^ transcripts under denaturing conditions. To further test whether the m.5783C > T mutation affects the conformation of tRNA^Cys^, total mitochondrial RNAs were electrophoresed through a 10% polyacrylamide gel (native condition) in Tris borate-EDTA buffer and then electroblotted onto a positively charged nylon membrane for hybridization analysis with oligodeoxynucleotide probes for tRNA^Glu^, tRNA^Leu(UUR)^ and tRNA^Met^, respectively. As shown in Figure 4C, electrophoretic patterns showed that the tRNA^Cys^ in three mutant cybrids carrying the m.5783C > T mutation migrated slower than those of three control cybrids lacking this mutation.

To further evaluate whether the m.5783C > T mutation impairs tRNA metabolism, we subjected mitochondrial RNAs from mutant and control cybrids to Northern blots and hybridized with DIG-labeled oligodeoxynucleotide probes for 8 tRNAs including tRNA^Cys^, tRNA^Tyr^ derived from the L-strand transcripts and 14 tRNAs including tRNA^Thr^, tRNA^Leu(UUR)^ and tRNA^Ser(AGY)^ derived from the H-strand transcripts as well as a nucleus-encoded 5S rRNA (under denaturing condition) (15,25). As shown in Figure 4D and Supplemental Figure S4, there was no migration difference in electrophoretic patterns of 22 tRNAs between mutant and control cybrids under denaturing condition. As shown in Figure 4D, the amount of tRNA^Cys^ and tRNA^Tyr^ in three mutant cell lines was markedly decreased compared with those in three control cell lines. For comparison, the average levels of each tRNA in the control or mutant cybrids were normalized according to the level of 5S rRNA. As shown in Figure 4E, the average levels of tRNA^Cys^ and tRNA^Tyr^ in three mutant cybrids were 55.6% (*P* < 0.001) and 61.2% (*P* < 0.001) of mean values of three control cybrids, respectively. However, the average levels of other 20 tRNAs in the mutant cybrids were comparable with those in control cybrids (Figure E, Supplemental Figure S4). The reductions in the levels of tRNA^Cys^ and tRNA^Tyr^ m.5783C > T mutation may be due to the impaired processing of tRNA^Cys^ precursors, as the adenine at position 5826 is the sharing nucleotide that acts as the 5′ end of tRNA^Cys^ and 3′ end of tRNA^Tyr^ genes, respectively (6,33).

### Shortened half-life of tRNA^Cys^

**Figure 5.**
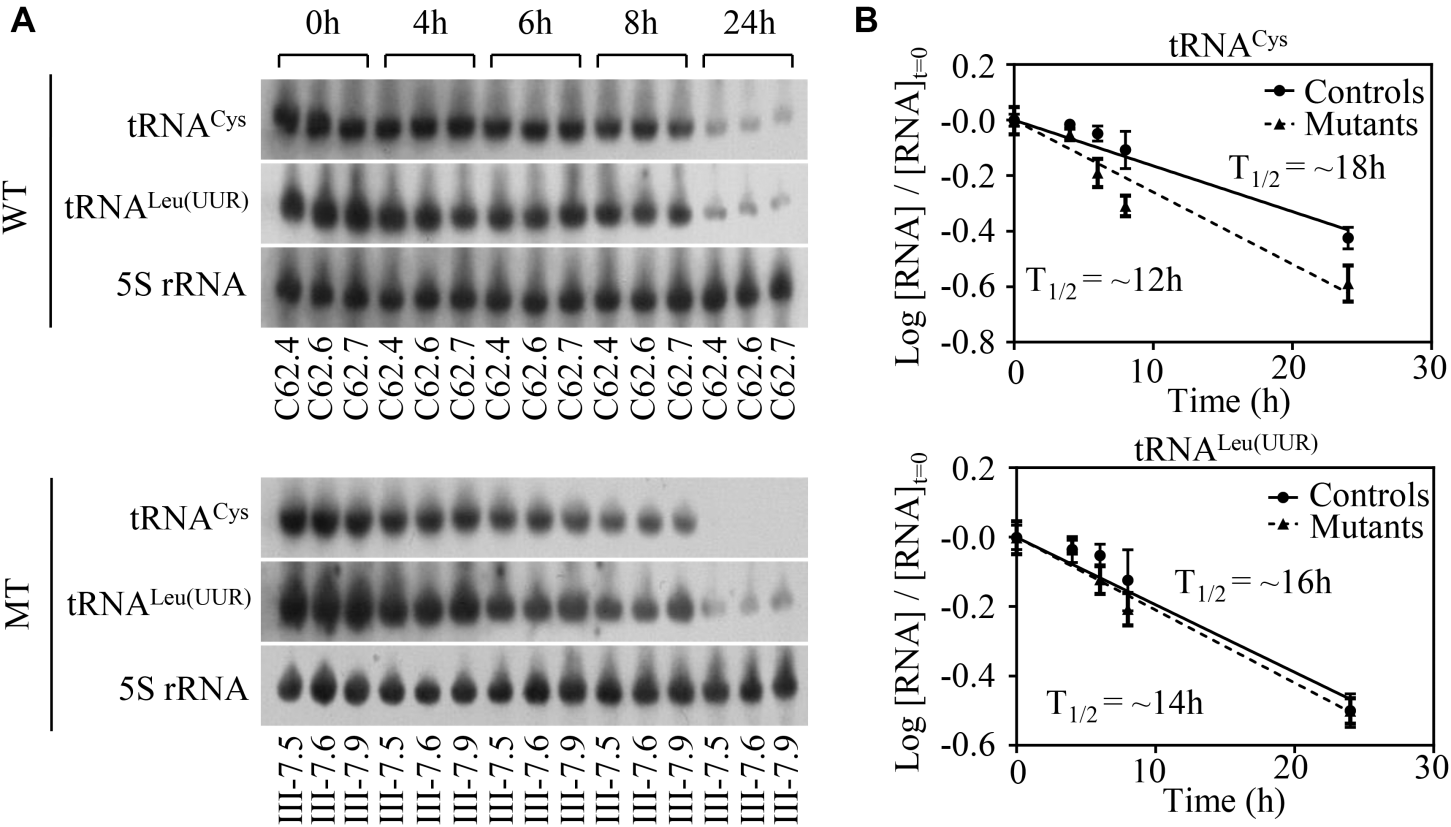
Decay kinetics of tRNA in three mutant cybrids and three control cybrids. (**A**) Mutant and control cell lines were treated with EtBr at various time courses. Total RNAs were isolated, electrophoresed through polyacrylamide gel, electroblotted, and hybridized with DIG-labeled oligonucleotide tRNA probes for tRNA^Cys^, tRNA^Leu(UUR)^ and 5S rRNA, respectively. (**B**) tRNA levels were measured using Image J and expressed as a fraction of the signal obtained from a panel of replicates taken at zero time (the time at which EtBr was added) were converted to logarithms on the assumption of first-order decay kinetics. All hybridization signals were normalized to 5S rRNA as the loading control. The data plotted represent the mean ± 2S.D. (error bars) of three independent experiments. Lines of best fit (least squares method) are shown, *R*^2^ for the two panels (from top left, tRNA^Cys^ to tRNA^Leu(UUR)^) being 0.981, 0.913, 0.978 and 0.976, respectively. Graph details and symbols are explained in the legend to Figure 2.

To further evaluate whether m.5783C > T mutation affected the stability of tRNA^Cys^, we treated the various cell lines with EtBr (250 ng/ml) to block mitochondrial RNA synthesis and extracted total cellular RNA at various time points after the addition of drug. We then subjected mitochondrial RNAs from various cell lines to Northern blot analysis and hybridized them with a DIG-labeled oligodeoxynucleotide probes specific for tRNA^Cys^, tRNA^Leu(UUR)^ and 5S rRNA as a loading control. As shown in Figure 5, the life span of tRNA^Cys^ were decreased as compared to those in control cybrids. For comparison, tRNA half-life was calculated through the best least-squares line fit. The average half-life of tRNA^Cys^ in three mutant cybrid cell lines were 66.6% (*P* = 0.001) of mean values of three control cybrids. However, there were no significant differences in the half-lives of tRNA^leu(UUR)^ between mutant and control cell lines. These results further supported that m.5783C > T mutation has a significant effect on the stability of tRNA^Cys^_._

### Impaired the 5′ end processing of tRNA^Cys^ precursor

**Figure 6.**
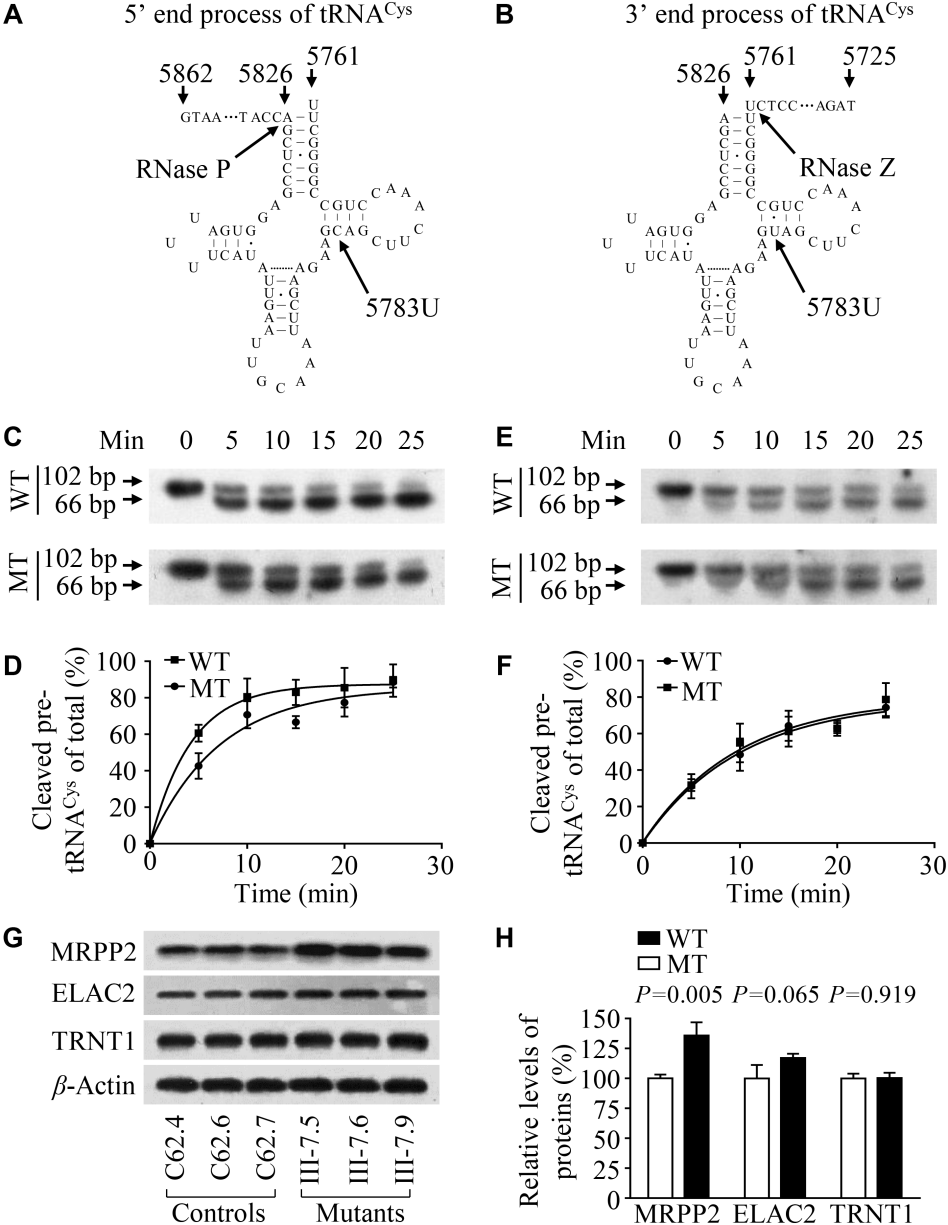
*In vitro* assay for the processing of mitochondrial tRNA^Cys^ precursors. (**A, B**) tRNA^Cys^ precursors for 5′ or 3′ end processing assays. Thirty-six nucleotides (nt) of 5′ and 3′ end leaders of tRNA^Cys^ were shown, including the m.5783C > T substitution. (**C**) *In vitro* 5′ end processing assays. Processing assays with mitochondrial RNase P were carried out in parallel for wild type and mutant substrates. Samples were withdrawn and stopped after 5, 10, 15, 20 and 25 min, respectively. Reaction products were resolved by denaturing polyacrylamide gel electrophoresis and reacted with a chemiluminescent substrate CDP-Star™ to detect the chemiluminescent. The graph shows the results of a representative experiment reaction. (**D**) Relative processing efficiencies of tRNA^Cys^ precursors catalyzed by RNase P. The relative processing efficiencies were calculated from the initial phase of the reaction. The calculations were based on three independent determinations. (**E**) *In vitro* 3′ end processing assays. Processing assays with mitochondrial RNase Z were undertaken in parallel for wild-type and mutant substrates. Samples were withdrawn and stopped after 5, 10, 15, 20 and 25 min, respectively. Reaction products were resolved by denaturing polyacrylamide gel electrophoresis and reacted with a chemiluminescent substrate CDP-Star™ to detect the chemiluminescent. The graph shows the results of a representative experiment reaction. (**F**) Quantification of the efficiencies of tRNA^Cys^ precursors catalyzed by RNase Z. The relative processing efficiencies were calculated from the initial phase of the reaction. The calculations were based on three independent determinations. (**G**) Western blot analysis of tRNA processing related proteins MRPP2, ELAC2 and TRNT1 with *β*-Actin as a loading control. (**H**) Quantification of MRPP2, ELAC2 and TRNT1. Average relative values of MRPP2, ELAC2 and TRNT1 were normalized to the average values of *β*-Actin in various cell lines. The values for the latter are expressed as percentages of the average values for the control cell lines. The average of three independent determinations for each cell line is shown. Graph details and symbols are explained in the legend to Figure 2.

We explored an in *vitro* processing system to assess the effect of m.5783C > T mutation on the 5′ end or 3′ end processing of tRNA^Cys^ precursors. For the 5′ end processing assays, the wild type and mutant tRNA^Cys^ precursors corresponding to mtDNA at positions 5761–5862 were incubated with mitochondrial RNase P, which was reconstituted from purified recombinant proteins MRPP1, MRPP2 and MRPP3 (Figure 6A), and precursors corresponding to mtDNA at positions 5725–5826 were incubated with RNase Z (Figure 6B) at various various time courses, respectively (16,17,25,30). No qualitative processing alterations of the mutant tRNA^Cys^ precursors were observed, but the 5' end processing efficiencies of the mutant tRNA^Cys^ transcripts were mildly decreased, as compared with those of wild-type counterparts (Figure 6C). The processing efficiencies of mutant tRNA^Cys^ transcripts catalyzed by RNase P were 59.7% of those in their wild-type counterparts (Figure 6D). However, there were no differences in the 3′ end processing efficiencies between mutant and wild-type counterparts (Figure 6E, F). As shown in Figure 6G and H, the effect of m.5783C > T mutation on 5′ end tRNA processing was further evidenced by upregulated MRPP2 (subunit of mitochondrial RNase P) in the mutant cybrids, as compared with control cybrids. However, there were no differences in the levels of ELAC2 (C terminal domain of RNase Z) and CCA-adding enzyme TRNT1 between mutant and control cybrids. These results demonstrated that the m.5783C > T mutation perturbed the 5′ end but not the 3′ end processing of tRNA^Cys^ precursors.

### Aberrant aminoacylation of tRNA^Cys^

**Figure 7.**
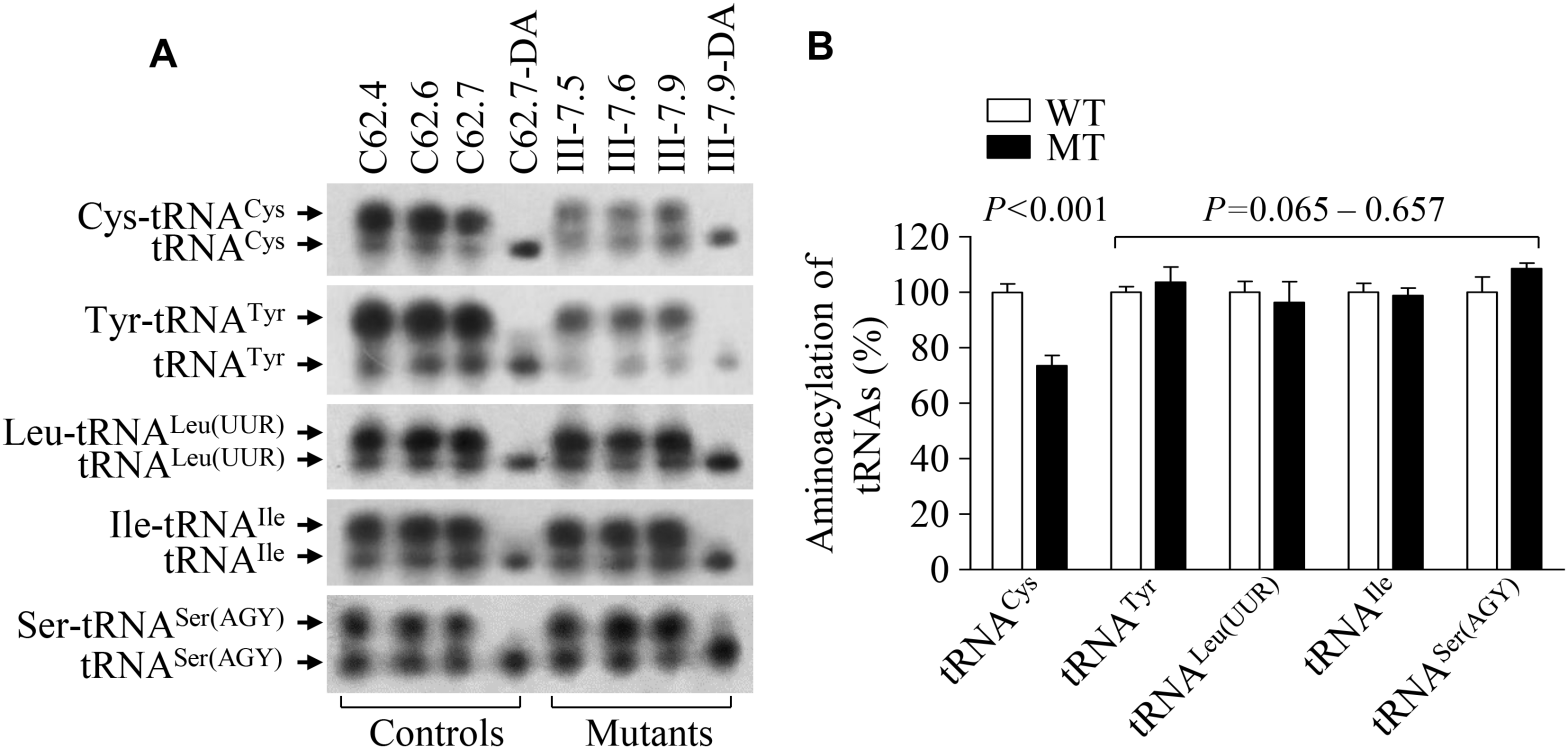
*In vivo* aminoacylation assays. (**A**) Ten micrograms of total cellular RNA purified from six cell lines under acid conditions were electrophoresed at 4°C through an acid (pH 5.0) 10% polyacrylamide-8 M urea gel, electroblotted, and hybridized with a DIG-labeled oligonucleotide probe specific for the tRNA^Cys^. The blots were then stripped and rehybridized with probes for tRNA^Tyr^, tRNA^Leu(UUR)^, tRNA^Ile^ and tRNA^Ser(AGY)^, respectively. The samples from one control (C62-7) and mutant (III-7.9) cell lines were deacylated (DA) by heating for 10 min at 60°C at pH 8.3 and electrophoresed as above. Aminoacylation assays for tRNA^Cys^ were carried out in parallel for aminoacylated and deacylated samples. (**B**) Qualification of aminoacylated proportions of tRNAs in the mutant and control cell lines. The calculations were based on three independent determinations. Graph details and symbols are explained in the legend to the Figure 2.

To investigate whether the m.5783C > T mutation affected the aminoacylation of tRNA^Cys^, we examined the aminoacylation properties of tRNA^Cys^, tRNA^Thr^, tRNA^Leu(UUR)^, tRNA^Ile^ and tRNA^Ser(AGY)^ by the use of electrophoresis in an acidic urea PAGE system to separate uncharged tRNA species from the corresponding charged tRNA, electroblotting and hybridizing with the tRNA probes as described above. As shown in Figure 7A, the upper and lower bands represented the charged and uncharged tRNA, respectively. The electrophoretic mobility of either charged or uncharged tRNA^Cys^ in mutant cell lines migrated slightly slower than those of control cell lines. To further distinguish nonaminoacylated tRNA from aminoacylated tRNA, samples of tRNAs were deacylated by being heated for 10 min at 60°C at pH8.3 and then run in parallel. Only one band (uncharged tRNA) was present in both mutant and control cell lines after deacylating. As shown in Figure 7B, the efficiencies of aminoacylated tRNA^Cys^ in three mutant cell lines were 73.5% of the average values of three control cell lines, while there were no obvious differences in the electrophoretic mobility and levels of tRNA^Thr^, tRNA^Leu(UUR)^, tRNA^Ile^ and tRNA^Ser(AGY)^ between mutant and control cell lines.

### Impairment of mitochondrial protein synthesis

**Figure 8.**
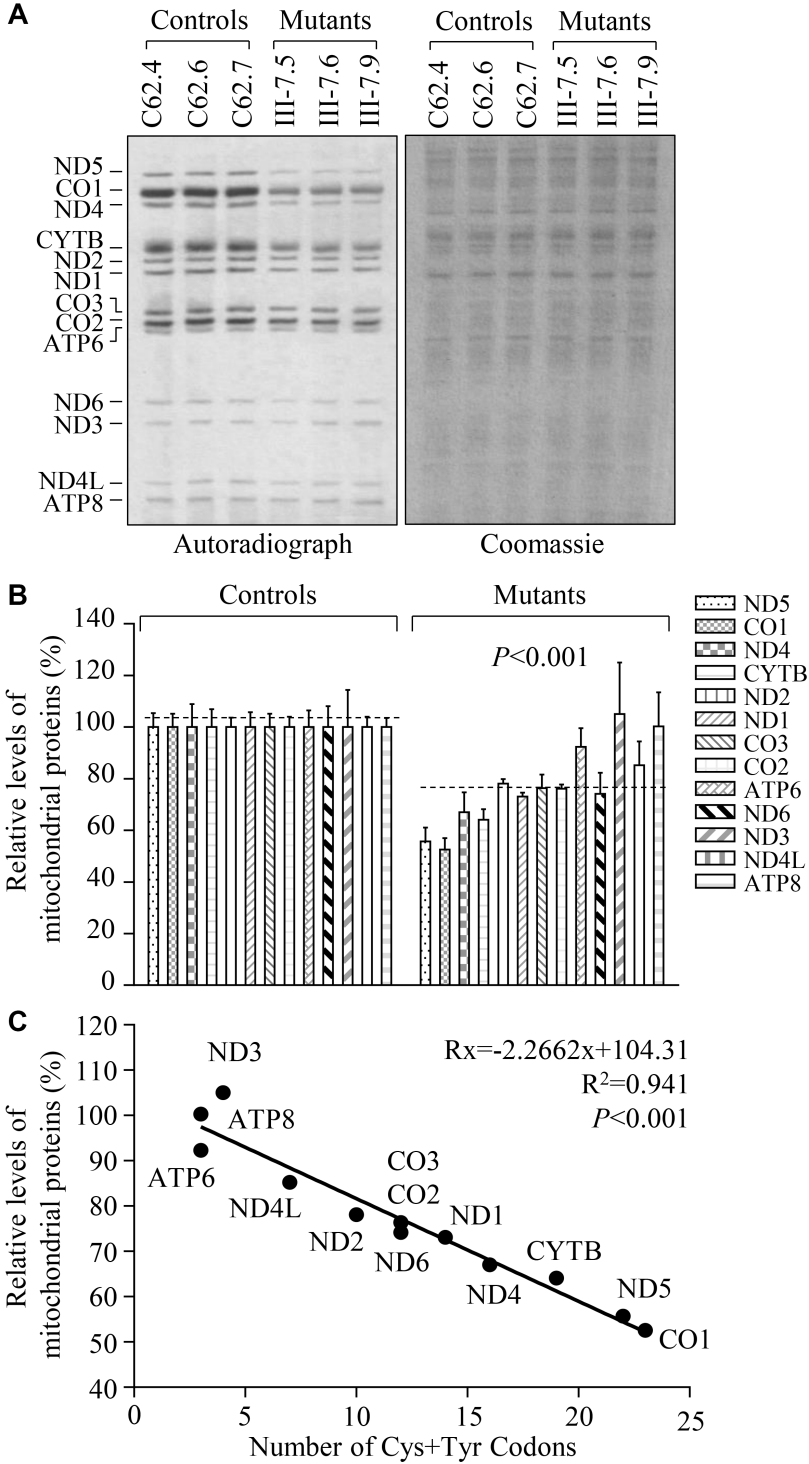
Analysis of mitochondrial translation. (**A**) Representative gel for electrophoretic patterns of the mitochondrial translation products of six cell lines labeled for 30 min with [^35^S]-methionine in the presence of 100 μg/ml of emetine and corresponding Coomassie Brilliant Blue stained gel used as loading controls. Samples containing equal amounts of proteins (30 μg) were run in SDS/polyacrylamide gradient gels. CO1, CO2 and CO3 indicate subunits I, II and III of cytochrome c oxidase; ND1, ND2, ND3, ND4, ND4L, ND5 and ND6, subunits 1, 2, 3, 4, 4L, 5 and 6 of the respiratory chain reduced nicotinamide-adenine dinucleotide dehydrogenase; ATP6 and ATP8, subunits 6 and 8 of the H^+^-ATPase; and CYTB, apocytochrome *b*. (**B**) Quantification of the rates of mitochondrial translation labeling. The rates of mitochondrial protein labeling in the mutant cybrids were expressed as percentages of the value for average values of three control cybrids in each gel. (**C**) Relationship between average relative levels of synthesis of the 13 polypeptides in the mutant cell lines and the number of cysteine plus tyrosine codons. Lines of best fit (least squares method) are shown, *R*^2^= 0.941. The curve shown describes the equation: *R^x^* = −2.2662*x* + 104.31, whose parameters have been optimized to make the best fit to the data. *R^x^* is the level of labeling of a polypeptide having x number of Cys + Tyr residues in mutant cell lines relative to the level in control cell lines. Graph details and symbols are explained in the legend to Figure 2.

To examine whether the aberrant tRNA metabolism caused by m.5783C > T mutation impaired mitochondrial translation, mutant and control cybrids were labeled for 30 min with ^[35S]^methionine-^[35S]^cysteine in methionine-free regular DMEM medium in the presence of 100 μg/ml of emetine to inhibit cytosolic protein synthesis (51). Figure 8A showed typical electrophoretic patterns of translation products from mutant and control cell lines. Patterns of mtDNA-encoded polypeptides of mutant cybrids harboring the m.5783C > T mutation were qualitatively identical to those of the control cybrids, in terms of electrophoretic mobility of the various polypeptides. However, cell lines carrying the m.5783C > T mutation displayed significant decreases in the total rate of labeling of mitochondrial translation products relative to those of the control cell line. As shown in Figure 8B, the overall levels of 13 mitochondrial translation products in three mutant cybrids were 76.9%, relative to the mean values measured in three control cybrids. In particular, the average levels of ND1, ND2, ND3, ND4, ND4L, ND5, ND6, CYTB, CO1, CO2, CO3, ATP6 and ATP8 in the mutant cybrids were 73.1%, 78.1%, 105.0%, 67.1%, 85.2%, 55.7%, 74.2%, 64.1%, 52.6%, 76.1%, 76.4%, 92.3% and 100.3% of those in the control cybrids, respectively.

As shown in Supplemental Table S3, the reductions in labeling of various polypeptides did not vary in relationship to the number or proportion of cysteine or tyrosine codons or proportion of cysteine + tyrosine codons in the corresponding mRNAs. As shown in Figure 8C, the experimentally determined labeling rates of all polypeptides, when related to the number of cysteine + tyrosine codons in the corresponding mRNAs, conformed well (*P* < 0.001) to the equation *R^x^* = −2.2662*x* + 104.31, whose parameters have been optimized to make the best fit to the data. *R^x^* is the level of labeling of a polypeptide having *x* number of Cys + Tyr residues in the mutant cybrids relative to the level in the control cybrids.

### Alterations in the stability and activities of OXPHOS complexes

**Figure 9.**
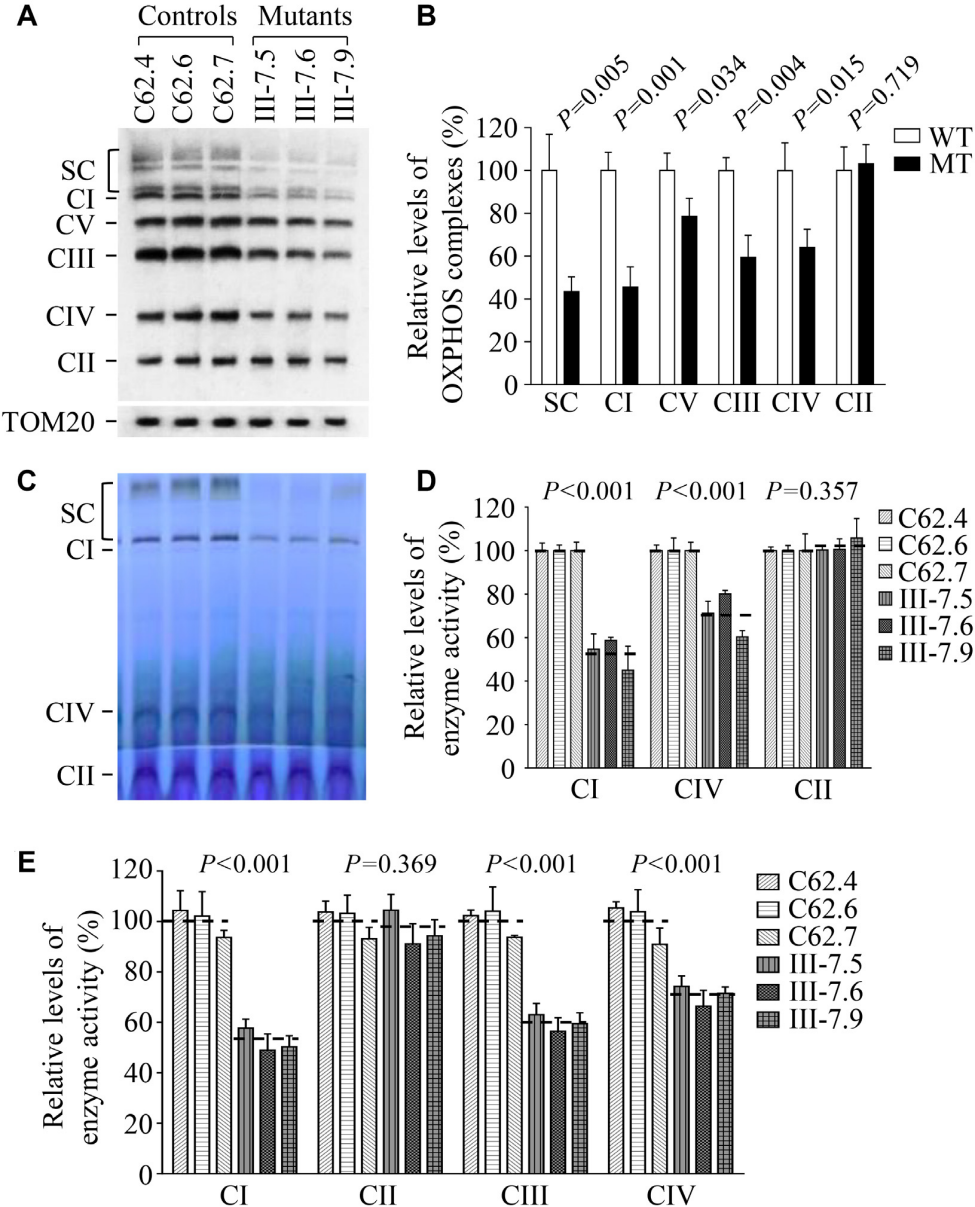
Analysis of OXPHOS complexes. (**A**) The steady-state levels of five OXPHOS complexes by Blue-Native gel electrophoresis. Thirty micrograms of mitochondrial proteins from mutant and control cell lines were electrophoresed through a Blue-Native gel, electroblotted and hybridized with antibody cocktail specific for subunits of each OXPHOS complex as well as Tom20 as a loading control. (B) Quantification in the levels of complexes I, II, III, IV, V and supercomplexes (SC) in mutant and control cell lines. The calculations were based on three independent experiments. (**C**) In-gel activity of complexes I, II and IV. Twenty micrograms of mitochondrial proteins from various cell lines were used for BN-PAGE, and the activities of complexes were measured in the presence of specific substrates (NADH and NTB for complex I, sodium succinate, phenazine methosulfate, and NTB for complex II, and DAB and cytochrome c for complex IV). (**D**) Quantification of in-gel activities of complexes I, II and IV. The calculations were based on three independent determinations in each cell line. (**E**) Enzymatic activities of respiratory chain complexes. The activities of respiratory complexes were investigated by enzymatic assay on complexes I, II, III and IV in mitochondria isolated from various cell lines. The calculations were based on four independent determinations in each cell line. Graph details and symbols are explained in the legend to Figure 2.

We examined the consequence of the m.5783C > T mutation on the assembly and activities of OXPHOS complexes. Mitochondria isolated from various cell lines were separated by blue native polyacrylamide gel electrophoresis (BN-PAGE) and Western blot analysis (52–54). As illustrated in Figure 9A, the mutant cell lines carrying m.5783C > T exhibited aberrant assembly of intact supercomplexes and complex I, III, IV and V, respectively. As shown in the Figure 9B, the average levels of supercomplexes (SC), complexes I (CI), complex III (CIII), complex IV (CIV) and complex V (CV) in three mutant cybrids were 43.46%, 45.50%, 78.55%, 59.41% and 64.09% of those in three control cybrids after normalization to TOM20, respectively. However, the levels of complex II (CII) in mutant cell lines were comparable with those in control cell lines. The lower levels of the respiratory complexes I, III and IV may be due to the misfolded and/or misassembled these complexes (53).

We then analyzed the stability and activities of complexes I, II and IV using the in-gel activity assay. Mitochondrial membrane proteins isolated from mutant and control cybrids were separated by BN-PAGE and stained with specific substrates of complexes I, II and IV (47,54). Defective assembly of intact supercomplexes and complexes I, IV were further confirmed in mutant cybrids carrying m.5783C > T mutation, as compared with control cybrids (Figure 9C and D). In particular, the average in-gel activities of complexes I and IV of three mutant cybrids were 54.64% and 71.18%, relative to the average values of three control cybrids, respectively. In contrast, the average in-gel activities of complexes II in these mutant cybrids were comparable with those of those control cybrids.

To further evaluate the effect of the m.5783C > T mutation on oxidative phosphorylation, we measured the activities of respiratory chain complexes by the use of isolating mitochondria from mutant and control cybrids. The activity of complex I (NADH ubiquinone oxidoreductase) was determined through the oxidation of NADH with ubiquinone as the electron acceptor (55,57). The activity of complex II (succinate ubiquinone oxidoreductase) was examined by the artificial electron acceptor DCPIP. The activity of complex III (ubiquinone cytochrome *c* oxidoreductase) was measured through the reduction of cytochrome *c* by using D-ubiquinol-2 as the electron donor. The activity of complex IV (cytochrome c oxidase) was monitored through the oxidation of cytochrome *c*. As shown in Figure 9E, the average activities of complexes I, III, and IV in three mutant cybrids were 52.35%, 59.65% and 70.68% of the mean values measured in three control cybrids, respectively, while the average activity of complex II in three mutant cybrids was comparable with those in three control cybrids.

## DISCUSSION

In this study, we demonstrated the profound impact of deafness-associated m.5783C > T mutation on mitochondrial DNA replication and tRNA metabolism contributing to the pathological process of deafness. The m.5783C > T mutation resides at a highly conserved cytosine at position 50 (C50) of TΨC stem of tRNA^Cys^ and adjacent to 5′ end of OriL spanning mtDNA positions at 5721–5798 containing the 37 bp of 3′ end anti-sense strand of tRNA^Cys^ gene, 32 bp noncoding sequence and 9 bp of 5′end anti-sense strand of tRNA^Asn^ gene (34–36). Human OriL consists of a stem-loop structure comprising a 12 nt loop and an 11 bp stem carrying RNA primer initiation for binding POLRMT and 5′end flanking sequences for L-strand replication (34,35,58–60). In the SDM model, replication initiates at OriH and continues around approximately two-thirds of the genome (∼11 kb) until reaching OriL that a single-stranded stem-loop structure is formed, whereby L-strand synthesis commences in the opposite direction, resulting in unidirectional fork progression from each distinct origin (8–11). Therefore, it was hypothesized that the m.5783C > T mutation affected L-strand mtDNA replication. *In vitro* replication analysis showed that the substitution of C to T at position 5783 reduced the efficiency of the L-strand replication (34). In the present study, neutral 2D agarose gel electrophoresis revealed significant reductions in the replication intermediates including marked decreases in the ascending arm of Y-fork arcs in 4 kb *Bcl*I fragment (nt 3658–7657) spanning OriL and mild reductions in the fork arcs in the 3 kb *Apa*I fragment (nt 1446–4431) in the mutant cybrids. By contrast, 2.8 kb *Acc*I fragment (nt 15 255−1504) spanning OriH and 1.85 kb *Dra*I fragment (nt 10 417–12 271) revealed similar patterns of mtDNA replication intermediates that formed archetypal bubble arcs in the *Acc*I fragment and fork arcs in the *Dra*I fragment between mutant and control cybrids. Southwestern blot analysis in the presence BrdU showed mild reductions in the amount of newly synthesized mtDNA in the mutant cybrids bearing m.5783C > T mutation, as compared with the control cybrids. The mtDNA replication defects were further evidenced by ∼50% decreases in the levels of components of mtDNA replication machinery: PolγA, Twinkle and SSBP1 in the mutant cybrids. In fact, alterations in the Twinkle, SSBP1 and polymerase POLG caused distinct replication stalling phenotypes and reduced mtDNA copy numbers (60–62). In this study, both Southern blot and qPCR assays showed that the levels of mtDNA contents in mutant cybrids exhibited progressive decreases until 50% during culturing first 7 weeks, but not further reductions until 14 weeks, as compared with control cybrids. These data suggested that 50% reductions in mtDNA contents may be the threshold level for respiration defects in the mutant cybrids bearing m.5783C>T mutation. The decreases in mtDNA contents were likely due to the slower mtDNA replication caused by the m.5783C > T mutation. These data demonstrated that the m.5783C > T mutation impaired the mtDNA replication, specifically for reducing efficiencies of L-strand replication.

The m.5783C > T mutation destabilized the canonical C50-G63 base-pairing within TΨC stem of mitochondrial tRNA^Cys^. Thus, we hypothesized that the m.5783C > T mutation impacted the structure and function of this tRNA, including alterations in the conformation, processing of tRNA precursors, stability and aminoacylation. In fact, the *T*_m_ in mutant tRNA^Cys^ molecule was 2°C lower than those in the WT counterpart. The instability of the mutant tRNA molecule was further evidenced by the drastically reduced levels and half-life of tRNA^Cys^ in the mutant cell lines carrying the m.5783C > T mutation, as in the case of tRNA^His^ 12201T > C mutation (50). Furthermore, the m.5783C > T mutation caused the conformational change of tRNA^Cys^, suggested by slower electrophoretic mobility of mutated tRNA with respect to the WT molecule *in vitro* or *ex vivo*, consistent with the conformational changes of tRNA^Glu^ carrying the m.14692A > G mutation (29). Notably, the mutant cybrids carrying the m.5783C > T mutation revealed significant decreases in the level of tRNA^Tyr^ but not those in tRNA^Asn^ and other tRNAs. On the L-strand transcripts, an adenine at position 5826 is a sharing nucleotide that acts as the 5′ end of tRNA^Cys^ and 3′ end of tRNA^Tyr^, while there was a 32 bp noncoding sequence junction between tRNA^Cys^ and tRNA^Asn^ (6). The *in vitro* processing assays revealed that the m.5783C > T mutation impaired the 5′ end but not the 3′ end processing of tRNA^Cys^ precursors. Hence, impaired 5′ end processing of tRNA^Cys^ caused by m.5783C > T mutation contributed to the decreased levels of tRNA^Cys^ and tRNA^Tyr^ but did not affect the other tRNAs on the L-strand transcripts. These data were in contrast with the observations that the aberrant 5′ end processing of tRNA^Ser(UCN)^ caused by m.7516DelA mutation yielded the decreased levels of tRNA^Ser(UCN)^ and downstream 5 tRNAs, and of tRNA^Gln^ caused by m.4401A > G mutation resulted in reduced levels of all 8 tRNAs and ND6 mRNA from the L-strand transcripts (15,25). Moreover, the cell lines bearing the m.5783C > T mutation displayed reduced aminoacylation efficiency of tRNA^Cys^, with respect to the wild-type cell lines, comparable with those in cell lines harboring 59A >G mutation at T-loop of tRNA^Ile^ (30). Therefore, the pleiotropic effects of m.5783T > C mutation on tRNA metabolism including the 5′ end processing of tRNA^Cys^ precursor, stability and aminoacylation of tRNA^Cys^ contributed to marked reductions in the steady-state levels of tRNA^Cys^ and tRNA^Tyr^.

The m.5783C > T mutation-induced alterations in mtDNA replication and tRNA metabolism resulted in impairment of mitochondrial translation and subsequently deficient oxidative phosphorylation. In this study, variable decreases (an average decreases of ∼23%) in 13 mtDNA-encoded polypeptides were observed in the mutant cybrids carrying the m.5783C > T mutation, comparable effects seen in cells bearing the m.4401A > G or m.7516DelA mutation (15,25). Strikingly, mutant cybrids carrying the m.5783C > T mutation revealed marked reductions (53% to 67%) in the levels of 4 polypeptides (ND4, ND5, CO1 and CYTB) harboring higher numbers of cysteine and tyrosine codons. By contrast, the levels of ND6, ATP6 and ATP8 with lower numbers of cysteine and tyrosine codons in mutant cybrids were comparable with those in control cybrids. As shown in the Supplementary Table S3, the reduced levels of these polypeptides in mutant cybrids were significantly correlated with the numbers of cysteine and tyrosine, in agreement with previous study in cells carrying the tRNA^Lys^ 8344A > G mutation (63). The impaired synthesis of mtDNA encoding subunits of OXPHOS gave rise to the instability of complexes I, III, IV and V as well as intact supercomplexes observed in the mutant cell lines carrying m.5783C > T mutation. In fact, each OXPHOS complex comprised of mtDNA-encoded subunit(s) and nuclear-encoded subunits, except complex II (64). These mtDNA-encoded subunits appear to act as seeds for building new complexes, which requires nuclear-encoded subunit import and assembly with the assistance of assembly factors (64). As a consequence, these defects yielded the reduced activities of these respiratory chain enzyme complexes. In particular, the impaired synthesis of ND4, ND5, CO1 and CYTB and CO3 was responsible for the decreased activities of complexes I, III and IV, respectively. The resultant respiratory deficiencies diminished mitochondrial ATP production and membrane potential, increased the ROS production and induced mitochondrial-dependent apoptotic death (25,30,50). These mitochondrial dysfunctions may lead to damaged or deficient inner ear hair cells that are particularly vulnerable to neurodegeneration related to oxidative phosphorylation, thereby contributing to the development of hearing loss (65–67). The hearing specific phenotypes of this tRNA mutation may be attributed to the tissue-specificity of OXPHOS via tRNA metabolism or modulation of nuclear modifier genes (68–71).

In summary, we demonstrated the profound impact of deafness-associated m.5783C > T mutation on mitochondrial DNA replication and tRNA metabolism contributing to the pathological process of hearing loss. The m.5783C > T mutation altered the mtDNA replication, specifically for reducing efficiencies of L-strand replication. The m.5783C > T mutation led to profound impact on tRNA metabolism including the instability, deficient aminoacylation and aberrant 5′ end processing of tRNA precursors. These failures in mtDNA replication and tRNA metabolism resulted in impairment of mitochondrial translation and subsequently deficient oxidative phosphorylation necessary for hearing function. Our findings provide new insights into the pathophysiology of maternal transmission of deafness arising from defects in mitochondrial DNA replication and tRNA metabolism.

## DATA AVAILABILITY

The authors declare that [the/all other] data supporting the findings of this study are available within the article [and its supplementary information files].

## Supplementary Material

gkac720_Supplemental_File
