# Supplementary material for: A deafness-associated mitochondrial DNA mutation caused pleiotropic effects on DNA replication and tRNA metabolism
Source: Nucleic Acids Res. 2022 Aug 30;50(16):9453–69. doi: 10.1093/nar/gkac720 (PMC9458427; doi:10.1093/nar/gkac720)
Supplement: gkac720_Supplemental_File [file gkac720_supplemental_file.pdf]

Supplemental Figure S1

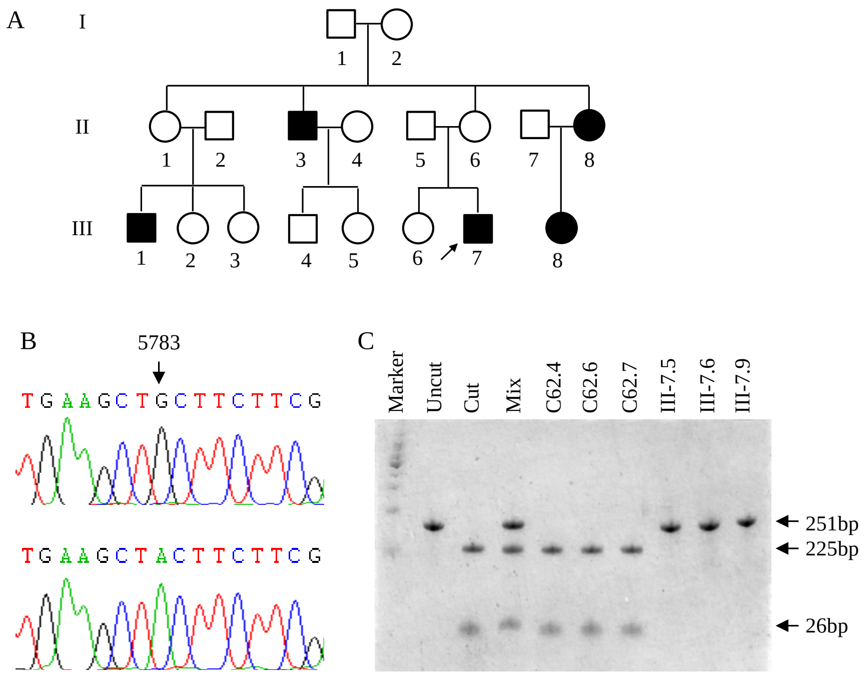

Supplemental Figure S1. Identification and qualification of m.5783C>T mutation. (A) One Han Chinese pedigrees with nonsyndromic hearing impairment. Hearing impaired individuals are indicated by filled symbols. Arrowhead denotes individuals that the lymphoblastoid cells derived from. (B) Partial sequence eletropherograms of the $tRNA^{Cys}$ gene from the cybrid cell lines of an affected individual (WZD117 III-7) and a Han Chinese control (C62), respectively. (C) Quantification of the m.5783C>T mutation by PCR-RFLP. PCR products were digested with *Pvu*II and analyzed by electrophoresis in a 10% polyacrylamide gel stained with ethidium bromide.

Supplemental Figure S2

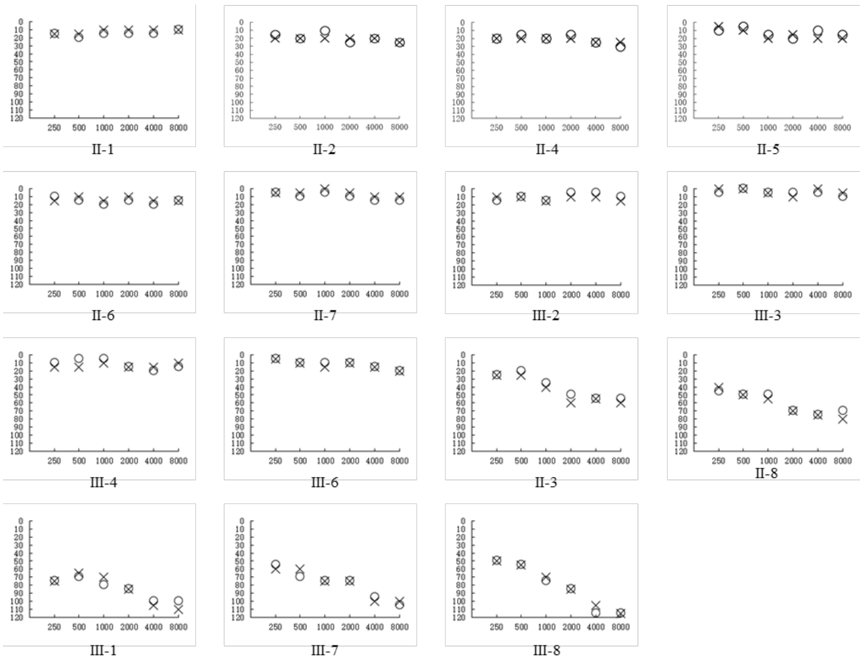

Supplemental Figure S2. Air conduction audiograms of some members of one Han Chinese family. X, left ear; O, right ear. An age-appropriate audiological examination was performed, and this examination included pure tone audiometry and/or auditory brainstem response, immittance testing and distortion product otoacoustic emissions. The pure tone audiometry was calculated from the sum of the audiometric thresholds at 500, 1000, 2000, 4000 and 8000 Hz. The severity of hearing impairment was classified into five grades: normal <26 dB, mild 26–40 dB, moderate 41–70 dB, severe 71–90 dB and profound >90 dB.

Supplemental Figure S3

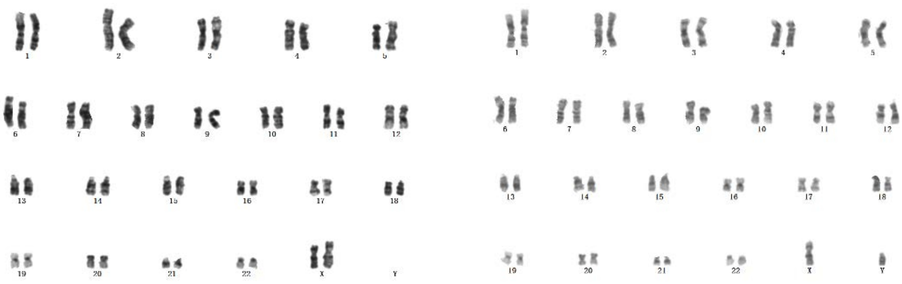

C62.4 III-7.5

Supplemental Figure S3. Karyotype analysis of control cybrid (C62.4) and mutant cybrid (III-7.5). Cyrids were incubated in DMEM medium with 0.2 µg/mL colcemid (Sangon) for 4 h. Cells were trypsinized and resuspended with 75 mM KCl at 37°C for 5 min. Cells were collected and fixed twice with a fixative composed of methanol and acetic acid (3:1, v/v) for 30 min. The cell suspension was dropped on the slides placed in chromosome distributor (THERMOTRON, Holland, MI, USA) and incubated for 5 min. The slides were incubated for 90 min at 80 °C, treated with trypsin for 1 s, and stained with Giemsa (Sangon) for 5 min. After washing in water, the slides were observed under oil-immersion microscopy.

Supplemental Figure S4

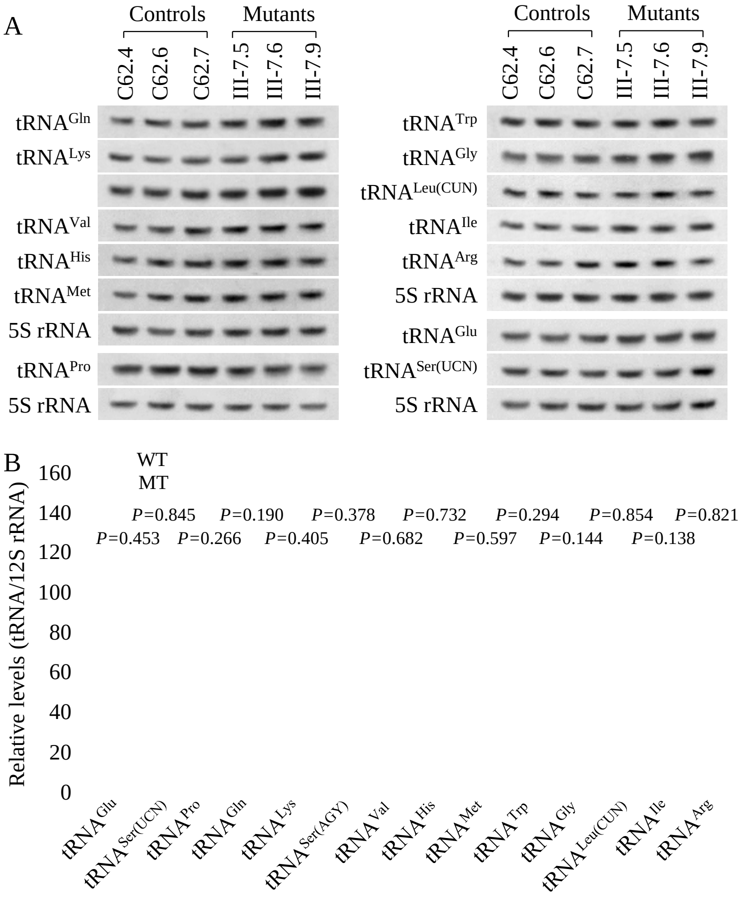

Supplemental Figure S4. Northern blot analysis of mitochondrial tRNAs. (A) Two microgram of total mitochondrial RNA from the various cell lines were electrophoresed through a 10% denaturing polyacrylamide gel, electroblotted and hybridized with DIG-labeled oligonucleotide probes specific for $tRNA^{Glu}$, $tRNA^{Ser(UCN)}$, $tRNA^{Pro}$, $tRNA^{Gln}$, $tRNA^{Lys}$, $tRNA^{Ser(AGY)}$, $tRNA^{Val}$, $tRNA^{His}$, $tRNA^{Met}$ $tRNA^{Trp}$ $tRNA^{Gly}$ $tRNA^{Leu(CUN)}$, $tRNA^{Ile}$ and 5S rRNA, respectively. (B) Quantification of the tRNA levels. Average relative each tRNA content per cell was normalized to the average content per cell of 5S rRNA in the control and mutant cybrids, respectively. The values for the latter were expressed as percentages of the average values for the control cybrids. The calculations were based on three independent determinations in each cybrids. The error bars indicate two standard errors of the mean. *P* indicates the significance, according to the *t*-test, of the difference between mutant and control cybrids.

Supplemental Table S1. Summary of the clinical and molecular data of some maternal members of a Chinese family

Subject	Gender	Age at test (years)	Age at onset (years)	PTA (dB) on right ear	PTA (dB) on left ear	Audiometric configuration	Level of hearing impairment	m.5783C>T mutation
II-1	F	61	-	12	15	-	Normal	-
II-2	M	64	-	21	19	-	Normal	+
II-3	M	58	46	44	40	slop	Moderate	-
II-4	F	58	-	22	21	-	Normal	+
II-5	M	55	-	15	13	-	Normal	+
II-6	F	51	-	13	16	-	Normal	-
II-7	M	47	-	9	10	-	Normal	+
II-8	F	47	43	62	60	slop	Moderate	-
III-1	M	36	27	85	85	slop	Severe	-
III-2	F	32	-	16	16	-	Normal	-
III-3	F	29	-	12	10	-	Normal	-
III-6	F	30	-	13	12	-	Normal	-
III-7	M	26	23	78	79	slop	Severe	-
III-8	F	21	20	80	83	slop	Severe	-

M, male; F, female; PTA, pure tone audiometry

Supplemental Table S2. mtDNA variants in one proband (WZD117-III-7) and one control individual (C62)

	Position	Replacement	Conservation (%) (17 species)*	CRS†	C62	WZD117-III-7)
D-loop	73	A to G		A	G	G
	263	A to G		A	A	G
	514	C Del		C	C	Del
	515	A Del		A	A	Del
	16157	T to C		T	C	T
	16129	G to A		G	G	A
	16180	A Del		A	A	Del
	16181	A Del		A	A	Del
	16182	A Del		A	A	Del
	16183	A Del		A	A	Del
	16184	C Del		C	C	Del
	16183	A to C		A	A	C
	16189	T to C		T	T	C
	16256	C to T		C	T	C
	16304	T to C		T	C	T
	16355	A to G		A	G	A
	16390	G to A		G	G	A
	16519	T to C		T	T	C
12S rRNA	750	A to G	100	A	G	G
	951	G to A	82	G	A	G
	1438	A to G	100	A	G	G
16S rRNA	1762	A to G	82	A	G	A
	2706	A to G	88	A	G	G
ND1	3970	C to T		C	T	C
ND2	4703	T to C		T	T	C
	4769	A to G		A	G	G
	5093	T to C		T	T	C
	5201	T to C		T	T	C
$tRNA^{Cys}$	5783	G to A	100	G	G	A
CO1	6446	G to A		G	A	G
	7028	C to T		C	T	C
CO2	7678	T to C		T	T	C
	8251	G to A		G	A	G
ATP6	8860	A to G (Thr to Ala)	82	A	G	G
	9177	A to G		A	A	G
ND4	11002	A to G		A	G	A
	11308	C to T		C	T	C
	11719	G to A		G	A	A
ND5	13928	G to C (Ser to Thr)	11	G	C	G
ND6	14227	C to T		C	T	C
CYTB	14766	C to T		C	T	T
	15326	A to G (Thr to Ala)	59	A	G	A
	15479	T to C (Phe to Leu)	82	T	C	T
	15734	G to A (Ala to Thr)	65	G	A	G

* Conservation of amino acid for polypeptides or of nucleotides for RNAs in 17 primary species including *Cebus albifron*, *Colobus guereza*, *Gorilla gorilla*, *Homo sapiens*, *Hylobates lar*, *Lemur catta*, *Macaca mulatta*, *Macaca sylvanus*, *Nycticebus coucang*, *Pan paniscus*, *Pan troglodytes*, *Papio hamadryas*, *Pongo pygmaeus*, *Pongo pygmaeus abelii*, *Tarsius bancanus*, *Trachypithecus obscurus*, *Chlorocebus sabaeus*; †CRS: Cambridge reference sequence;

Supplemental Table S3. Usage of cysteine and tyrosine codons in human mitochondrial genes and average levels of individual polypeptide in mutant cells related to the average values in the control cells.

Gene	No. of amino acids	Number (Cys)	proportion (Cys [%])	Number (Tyr)	Proportion (Tyr [%])	Number (Cys+Tyr)	Proportion (cys+Tyr [%])	Relative levels of polypeptides (%)
ND5	604	6	1.00%	16	2.60%	22	3.60%	55.68
CO1	514	1	0.20%	22	4.30%	23	4.50%	52.57
ND4	459	3	0.70%	13	2.80%	16	3.50%	67.06
Cytb	380	2	0.50%	17	4.50%	19	5.00%	64.13
ND2	347	0	0.00%	10	2.90%	10	2.90%	78.12
ND1	318	0	0.00%	14	4.40%	14	4.40%	73.07
CO3	261	1	0.40%	11	4.20%	12	4.60%	76.38
CO2	225	3	1.30%	9	4.00%	12	5.30%	76.10
ATP6	227	0	0.00%	3	1.30%	3	1.30%	92.33
ND6	175	1	0.60%	11	6.30%	12	6.90%	74.19
ND3	115	1	0.90%	3	2.60%	4	3.50%	105.04
ND4L	99	3	3.00%	4	4.00%	7	7.00%	85.24
ATP8	69	1	1.40%	2	2.90%	3	4.30%	100.29
